# Evidence-based green algal genomics reveals marine diversity and ancestral characteristics of land plants

**DOI:** 10.1186/s12864-016-2585-6

**Published:** 2016-03-31

**Authors:** Marijke J. van Baren, Charles Bachy, Emily Nahas Reistetter, Samuel O. Purvine, Jane Grimwood, Sebastian Sudek, Hang Yu, Camille Poirier, Thomas J. Deerinck, Alan Kuo, Igor V. Grigoriev, Chee-Hong Wong, Richard D. Smith, Stephen J. Callister, Chia-Lin Wei, Jeremy Schmutz, Alexandra Z. Worden

**Affiliations:** Monterey Bay Aquarium Research Institute, 7700 Sandholdt Rd, Moss Landing, CA 95039 USA; Biological Sciences Division, Pacific Northwest National Laboratory, Richland, WA 99352 USA; U.S. Department of Energy (DOE) Joint Genome Institute (JGI), Walnut Creek, CA 94598 USA; Hudson Alpha, 601 Genome Way, Huntsville, AL 35806 USA; Integrated Microbial Biodiversity Program, Canadian Institute for Advanced Research, Toronto, M5G 1Z8 Canada; Now at: Ronald and Maxine Linde Center for Global Environmental Science, California Institute of Technology, Pasadena, CA 91125 USA; Center for Research in Biological Systems and the National Center for Microscopy and Imaging Research, University of California, La Jolla, San Diego, California 92093 USA

**Keywords:** GreenCut, Archaeplastida evolution, Viridiplantae, Introner Elements, RNA sequencing, Proteomics, Evidence-based gene models, Peptidoglycan, PPASP

## Abstract

**Background:**

Prasinophytes are widespread marine green algae that are related to plants. Cellular abundance of the prasinophyte *Micromonas* has reportedly increased in the Arctic due to climate-induced changes. Thus, studies of these unicellular eukaryotes are important for marine ecology and for understanding Viridiplantae evolution and diversification.

**Results:**

We generated evidence-based *Micromonas* gene models using proteomics and RNA-Seq to improve prasinophyte genomic resources. First, sequences of four chromosomes in the 22 Mb *Micromonas pusilla* (CCMP1545) genome were finished. Comparison with the finished 21 Mb genome of *Micromonas commoda* (RCC299; named herein) shows they share ≤8,141 of ~10,000 protein-encoding genes, depending on the analysis method. Unlike RCC299 and other sequenced eukaryotes, CCMP1545 has two abundant repetitive intron types and a high percent (26 %) GC splice donors. *Micromonas* has more genus-specific protein families (19 %) than other genome sequenced prasinophytes (11 %). Comparative analyses using predicted proteomes from other prasinophytes reveal proteins likely related to scale formation and ancestral photosynthesis. Our studies also indicate that peptidoglycan (PG) biosynthesis enzymes have been lost in multiple independent events in select prasinophytes and plants. However, CCMP1545, polar *Micromonas* CCMP2099 and prasinophytes from other classes retain the entire PG pathway, like moss and glaucophyte algae. Surprisingly, multiple vascular plants also have the PG pathway, except the Penicillin-Binding Protein, and share a unique bi-domain protein potentially associated with the pathway. Alongside *Micromonas* experiments using antibiotics that halt bacterial PG biosynthesis, the findings highlight unrecognized phylogenetic complexity in PG-pathway retention and implicate a role in chloroplast structure or division in several extant Viridiplantae lineages.

**Conclusions:**

Extensive differences in gene loss and architecture between related prasinophytes underscore their divergence. PG biosynthesis genes from the cyanobacterial endosymbiont that became the plastid, have been selectively retained in multiple plants and algae, implying a biological function. Our studies provide robust genomic resources for emerging model algae, advancing knowledge of marine phytoplankton and plant evolution.

**Electronic supplementary material:**

The online version of this article (doi:10.1186/s12864-016-2585-6) contains supplementary material, which is available to authorized users.

## Background

**Fig. 1:**
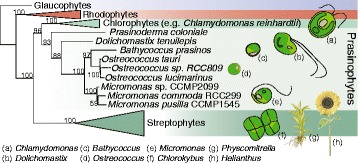
Relationships of photosynthetic eukaryotes in the Archaeplastida. The cladogram was inferred with the cpREV + G model using maximum likelihood methods and a concatenated alignment of 16 plastid-genome encoded proteins. Bootstrap support >50 % is shown. Plastid genome information is lacking for *O. lucimarinus* and RCC809, therefore, branching within the *Ostreococcus* clade is based on branching in the 18S rRNA gene tree [9] and is represented with dashed lines

Marine photosynthetic plankton are responsible for approximately half of global carbon fixation [1]. Prasinophytes are a major lineage of unicellular green algae that can contribute significantly to marine primary production [2–4]. In the oceans, prasinophyte genera within Class II are particularly widespread. These algae include the picoplanktonic (<2 μm cell diameter) genera *Bathycoccus*, *Micromonas* and *Ostreococcus* which have small genomes (12–22 Mb) and less gene family expansion than observed in other Viridiplantae groups (Fig. 1), specifically chlorophyte algae and streptophytes [5]. Like chlorophytes, prasinophytes provide insights into ancestral Viridiplantae gene families. For example, key transcription factors formerly considered innovations in vascular plants are present in *Micromonas* although absent from model chlorophytes, such as *Chlamydomonas reinhardtii* and non-vascular plants like *Physcomitrella patens* (moss) [6].

The first described eukaryotic picoplankter was *Chromulina pusilla* [7], later renamed *Micromonas pusilla. Micromonas* forms at least seven phylogenetically distinct clades, six of which have cultured representatives [8–10]. These clades appear to often co-exist in mid- to low- latitude systems [10, 11], with the exception of *Micromonas* Clade E2 which is found in polar environments but not lower latitude surface oceans [9]. Abundance of the latter has reportedly increased in the Canadian Arctic in association with climate induced changes [2]. Like *Micromonas*, the genus *Bathycoccus* is also found from tropical to polar systems, but is much less phylogenetically diverse [12, 13]. Their sister genus *Ostreococcus* is found only in mid- and low- latitude waters and has several established clades with distinct environmental distributions [14, 15].

Morphologically the three genera have marked differences. All have a single chloroplast and lack visible cell walls. Unlike *Bathycoccus* and other known prasinophytes, *Micromonas* and *Ostreococcus* do not have scales [16]. Additionally, *Bathycoccus* and *Ostreococcus* are non-motile while *Micromonas* has a flagellum (like most prasinophytes) and is larger than the former two taxa. Genomes have been sequenced for *Micromonas* species representing Clades D (*Micromonas pusilla* CCMP1545) and A (*Micromonas* RCC299) [6]. In addition, three *Ostreococcus* and one *Bathycoccus* species have completely sequenced genomes [17–19], while targeted *Bathycoccus* metagenomes have been sequenced from coastal Chile [13] and the tropical Atlantic Ocean [13, 20]. The *Micromonas* nuclear genomes are 22 Mb (CCMP1545) and 21 Mb (RCC299), while the genomes of *Bathycoccus prasinos* (15 Mb) and various *Ostreococcus* (~13 Mb) are smaller [6, 17–19]. Genomes of all three genera contain two chromosomes with lower GC% than the overall average (e.g., 51 % versus the overall average of 64–66 % in *Micromonas*). The larger low-GC region (LGC) is a proposed sex chromosome, while the other is much smaller and has few recognizable genes [6, 17, 19]. The RCC299 genome sequence is gapless, with telomere to telomere sequenced chromosomes [6]. In contrast the CCMP1545 genome was published as a high quality draft genome (Sanger sequenced) in 21 scaffolds representing 19 chromosomes.

To further develop genomic resources for Class II prasinophytes (the Mamiellophyceae), we finished sequences from four CCMP1545 chromosomes and developed new gene models for both CCMP1545 and RCC299 using evidence-based methods, including directional Illumina RNA-Seq and Liquid Chromatography Tandem Mass Spectrometry (LC-MS/MS) proteomics. Analyses of these datasets revealed characteristics of gene architecture, novel repetitive introns and deviations in splice donor sequence. We also analyzed the predicted proteome of polar Clade E2 isolate CCMP2099 and generated genomic information for a more basal Class II prasinophyte by growing and sequencing the transcriptome of *Dolichomastix tenuilepis*. Our comparative studies identified proteins that are likely involved in scale formation and features of the land plant ancestor as well as essential components of photosynthesis. Among these is the presence of a bacterial-like peptidoglycan pathway that has been retained in lineages from across the Archaeplastida supergroup (Fig. 1), but selectively lost in multiple independent events. Our studies highlight the complementarity of two evolutionary distinct green algal groups, represented by *Micromonas* and *Chlamydomonas*, for investigating plant systems and provide new insights into the development of the green lineage.

## Results and discussion

### Genome improvement and evidence-based gene models

**Fig. 2:**
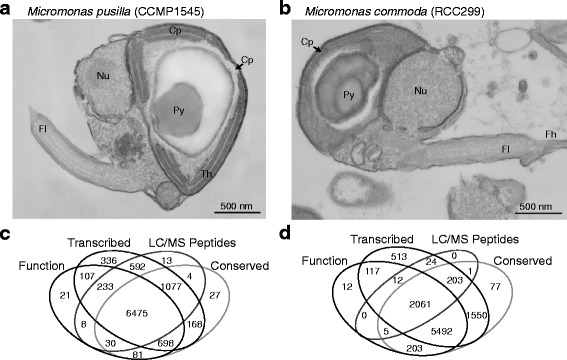
*Micromonas pusilla* and *M. commoda* cellular structures and gene model sets. Transmission electron micrographs of CCMP1545 (**a**) and RCC299 (**b**) show the chloroplast (Cp) comprising more than half the cell, the pyrenoid (Py), nucleus (Nu) and flagellum (Fl) with microtubules visible (24 nm diameter). In RCC299 the beginning of the flagellar hair (Fh) is also visible as are the thylakoid membranes in CCMP1545 (Th). Evidence for new gene models in CCMP1545 (**c**) and RCC299 (**d**) is shown based on: Function: Predicted protein had a functional domain hit in Interproscan; Transcribed: Predicted gene model was expressed (FPKM > 2,000) in the RNA-Seq experiment; LC-MS Peptides: At least one peptide matching the predicted protein was identified in the LC-MS/MS analysis; Conserved: Blastp hits were found in NCBI’s nr database (E-value ≤10^−15^). The total number of genes with no evidence: 25 (CCMP1545) and 37 (RCC299). Note that for RCC299 the lower number of gene models supported by LC-MS/MS data (2,306) than in CCMP1545 (8,432) is due to generation of fewer peptides

We finished four chromosomes in CCMP1545 which reduced the total number of gaps in the genome sequence from 582 to 455. Specifically, scaffolds 2, 3, 18 and 19 were finished by closing all gaps, improving low quality regions and resolving repeat structures. These four chromosomes now represent 4,888,335 base pairs (bp) of finished sequence (Additional file 1: Table S1). Of the other scaffolds in the initially published genome build [6], scaffolds 20 (8,981 nt) and 21 (5,431 nt) are not considered to be full chromosomes, because the former represents an unresolved repeat unit on scaffold 5 and the latter is rDNA. The newly finished Chromosome 19 is the smallest (0.25 Mb) in the genome, and has 51 % GC. Chromosome 2 contains the LGC (48 % GC), which spans 1.7 Mb of its total 2.2 Mb. The remainder of Chromosome 2 is 63 % GC, similar to the overall genome average (66 %, Additional file 2: Figure S1). The total chromosome count is 17 for RCC299 [6] and 19 for CCMP1545. We performed transmission electron microscopy on CCMP1545 and RCC299 which showed similar morphologies and structures, with the chloroplast occupying more than half the cell (Fig. 2a, b).

Two forms of biological evidence for protein-encoding genes were generated for each *Micromonas* species. Three hundred and twenty six million paired-end RNA-Seq reads were generated for each *Micromonas* isolate alongside 758,467 (CCMP1545) and 236,683 (RCC299) peptide spectra generated by LC-MS/MS. These forms of evidence were used to score, select and modify the best gene model for each locus from an “allgenes” model set generated using multiple prediction algorithms [6] and to generate new models at previously unannotated loci. The new CCMP1545 protein-encoding gene model set contains 680 fewer genes than the initially published Gene Catalog [6] (Table 1). The reduction was primarily due to merging of adjacent gene models according to new transcriptional evidence and resulted in longer average gene length. In RCC299, average protein-encoding gene size increased largely due to 5′ coding region extension (Table 1). Removal of unsupported RCC299 gene models, gene merging, and creation or addition of models based on new evidence resulted in a set that contained 251 more protein-encoding gene models than the prior Catalog [6]. Average exon size increased by 15 (CCMP1545) and 23 % (RCC299) because of additional untranslated region (UTR) sequence and because average coding length increased (Table 1). Overall, the new models were 29 (CCMP1545) and 21 % (RCC299) longer than the original models.

**Table 1:** Comparison of the evidence-based (EB) protein coding gene model sets predicted here for the nuclear genomes of *Micromonas pusilla* (CCMP1545) and *Micromonas commoda* (RCC299) and the original model sets (“Catalog”) published in Worden et al. 2009 [6]

	CCMP1545 EB set	Catalog CCMP1545	RCC299 EB set	Catalog RCC299
Protein coding genes (number)	9895	10575	10307	10056
Average transcript length (nt)	1799	1390	1808	1497
Average coding length (nt)	1653	1317	1663	1419
Introns (number)	11229	9531	5418	5650
Average intron length (nt)	180	187	152	163
Exons (number)	21060	20106	15651	15706
Average exon length (nt)	840	731	1182	958
Spliced genes (number)	5666	5311	3584	3688
Exons per multiple-exon gene	2.98	2.79	2.51	2.54
Total intergenic bases (Mb)	2.3	n.c.	1.7	n.c.
Total exonic bases (Mb)	17.6	n.c.	17.4	n.c.
Total intronic bases (Mb)	0.20	n.c.	0.78	n.c.
GC splice donors (%)	25.60	5.90	1.50	0.70

Note that for average transcript and coding lengths introns have been removed. For the EB sets gene characteristics were computed on 9826 (CCMP1545) and 10233 (RCC299) proteins, to which 69 and 74 models were later added, respectively, due to new RNA-seq support. Abbreviation: n.c., not computed

Four independent tests were used to assess gene models and validity of predicted proteins. Specifically, (1) conservation of predicted proteins was examined using blastp searches (E-value ≤10^−15^) against NCBI’s non-redundant protein set (nr); (2) transcription was verified using RNA-Seq data; (3) translation was verified using LC-MS/MS support; and (4) predicted function was characterized using Interproscan [21]. The vast majority (9,870) of CCMP1545 protein-encoding gene models had supporting evidence, while 25 did not (Fig. 2c). Ninety-eight percent of genes were confirmed using RNA-Seq, and 96 % were supported by at least two types of evidence. Of the 21 predicted proteins with only Interproscan evidence, 11 were related Sel-1 like repeat (SLR) proteins. For RCC299, 97 % of models were confirmed with RNA-Seq and 94 % were supported by at least two evidence types (Fig. 2d), while 37 gene models had no evidence. Twelve predicted proteins from mixed families of unknown function (two with zinc finger predictions) had only Interproscan support.

### Gene overlap

For both species, gene density is lower than average on the smallest chromosomes (which also exhibit lower than average GC %; Additional file 1: Table S1). Gene density is also lower in the LGC, but unlike the smallest chromosomes, LGC genes are often organized in convergent overlapping pairs (COPs). These have overlapping 3′ UTRs and relatively large intergenic distances to their respective upstream (5′) neighbors. In the CCMP1545 LGC, 316 of 591 genes occur as COPs, with an average intergenic distance of 1,255 (± 837) nucleotides (nt) between the COP and non-COP neighbors. The average intergenic distance on the other 18 chromosomes is 211 ± 398 nt. Similarly, in the RCC299 LGC, 242 of 738 genes occur in COPs, with intergenic distances of 898 ± 810 nt that contrast with the average for the other 16 chromosomes of 167 ± 238 nt. COP numbers are likely underestimated because we required EST evidence (directionally cloned cDNAs, Sanger sequenced) as validation of overlapping models. Visual inspection of the RNA-Seq evidence indicates that many more tail to tail overlaps occur among LGC genes. Overlap between protein-coding genes in eukaryotes has been suggested as a mechanism for reciprocal regulation [18, 22]. For single cell organisms such as *Micromonas*, physical separation of cytoplasmic biochemical pathways is only feasible through temporal regulation and indeed rhythmic patterns in gene expression have been found in *Ostreococcus* [23] and CCMP1545 [24]. Further studies are needed to establish whether reciprocal regulation of COPs provides a mechanism for temporal partitioning of cellular processes and expressional programs in unicellular organisms.

### Architectural and intronic novelties

CCMP1545 and RCC299 have two clear differences in gene architecture; both are related to intron characteristics. We identified numerous GC splice donors in CCMP1545 (25.6 %) that were largely absent in initial predictions. This is likely because most prediction programs require GT/AG splice donor/acceptor pairs while the short read sequence aligner used here [25] accommodates both GT and GC splice donors. To our knowledge, only the marine haptophyte alga *Emiliania huxleyi* has more GC splice donors (50 %) [26] than CCMP1545. Unlike CCMP1545, the 1.5 % GC splice donors in RCC299 (Table 1) is nearly identical to other Viridiplantae, such as the streptophytes *Arabidopsis thaliana* (1.5 %) and *Brassica rapa* (1.2 %) [27–29].

**Fig. 3:**
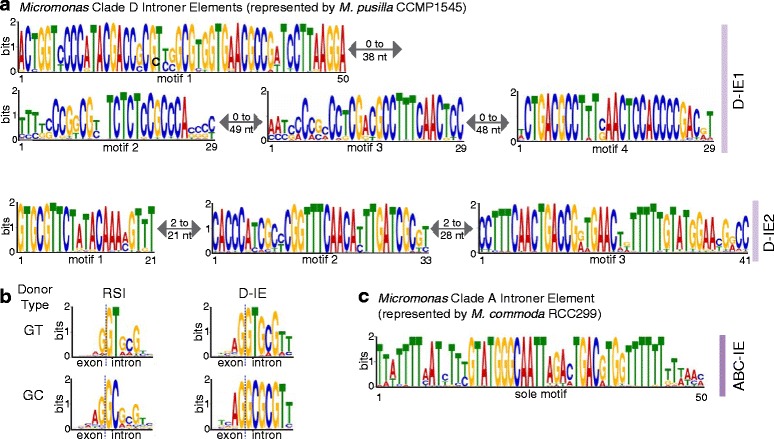
Introner Elements (IEs) and intron splice donor features. (**a**) D-IE1 and D-IE2 contain four and three motif groups, respectively. Minimum and maximum nucleotide stretches intervening between motifs are indicated. (**b**) CCMP1545 contains a high percentage of GC splice donors in IE containing introns (43.5 %) as well as regular spliceosomal introns (RSIs, 9.7 %). Splice donor motifs are shown for GT and GC motifs in both RSIs and IEs (motifs generated with 500 input sequences each). (**c**) The motif of the RCC299 ABC-IE (based on 164 ABC-IE containing introns)

CCMP1545 also has twice as many introns as RCC299, although both species contain similar numbers of nucleus-encoded genes (Table 1). Many of the introns in CCMP1545 are Introner Elements (IE), a type of spliceosomal intron that has recognizable branch points, but also has high sequence identity throughout the genome (unlike regular spliceosomal introns, RSIs) [6, 9, 30]. IE fragment the gene models produced by some prediction algorithms. To identify IE here, predicted introns in CCMP1545 were clustered to identify those with sequence similarity. A motif finder was used to identify sequence motifs; two groups of non-overlapping motifs were found: a four motif group that identified IE1, IE3, and IE4 as reported in (6), and a three motif group that recognizes IE2 (Fig. 3a). We will refer to these as D-IE1 and D-IE2 (exclusive to Clade D *Micromonas* [9]), respectively.

The non-overlapping motif sets had 0 to 49 nucleotides between those present in an intron (see Fig. 3a, Table 2) and were used to identify a total of 3,409 complete D-IE: 3,171 D-IE1 matched all of the motifs in the four motif group and 238 D-IE2 matched all the motifs in the three motif group. In addition to complete matches to the four motif (or three-motif) group, 3,131 partial D-IEs were identified. Among these, 572 matched three of the four D-IE1 motifs, 382 matched two D-IE1 motifs, and 1,501 matched only one D-IE1 motif (the first motif in 1,247 cases; Fig. 3a). Two full D-IE1s and one D-IE2 matched all four (or three) motifs, but with an internal duplication, possibly due to a merge of two originally complete D-IE1s. Partial D-IE2 elements were also found. These contained only two (287) motifs or just one (382, of which 354 are motif 1). In 18 of the 1,501 cases where a D-IE1 contained only one motif, that motif was present in multiple copies. Mixed-motif partials, with motifs from D-IE1 and D-IE2, occurred seven times. The large number of partial motifs inside introns indicates that both D-IE1 and D-IE2 are diverging from their source sequence and that much of the specific primary sequence of the IE is not necessary for splicing, supporting hypotheses put forth in [9].

**Table 2:** Introner Element families in *Micromonas* Clade D, as identified in CCMP1545, and in *Micromonas* Clades A, B, and C as identified in RCC299

IE family	D-IE1 (complete)	D-IE1 (partial)	D-IE2 (complete)	D-IE2 (partial)	ABC-IE
Motifs	4	1–3	3	1–2	1
Count	3171	2455	238	669	164
Intronic	3117	2194	201	337	164
Intergenic	25	46	32	303	0
GC splice donor	46 %	44 %	7 %	15 %	0 %

Sequences matching motifs from both IE1 and IE2 were omitted (7 total)

While the vast majority of D-IE are intronic and oriented in the same direction as the gene containing them, 34 intronic IEs appear to be on the opposite strand. Seven of these were complete D-IE (six D-IE1, one D-IE2). Additionally, 57 complete and 349 partial IE (according to our motif analysis) appear to be intergenic (Table 2). Some D-IE overlapped coding (153) or noncoding (131) exons, most of which (204) were located on the opposite strand. The majority (198 out of 284) were D-IE1 partials that only contained motif 1, suggesting possible integration into the CDS. Motif 1 does not encompass branch points, and is therefore unlikely to function as an independent intron. These deviations may also represent lingering issues with gene models or potentially other aspects of *Micromonas* IE dynamics and their proposed propagation at R-loops [9]. Overall, 5,850 D-IE (complete, partial, and mixed) are located in 5,499 introns of the new model set, 90 % of which are supported by spliced transcript data. Although 351 of these introns contain two or three IE (summing to six or more motifs), most IE containing introns have a single complete D-IE1.

Presence of D-IEs (complete and partial) is connected to higher percentages of GC splice donors, 79 % of which occur in D-IE1-containing introns. Still, 54 % of D-IE1 have GT splice donors, indicating that selection may act against the GC splice donor. Only 15 % of introns containing complete D-IE2 have GC splice donors, while introns that contain a full D-IE1 have GC splice donors 46 % of the time. Interestingly, sequence proximal to D-IE splice donors is much more conserved than for RSIs, regardless of GC or GT donor state (Fig. 3b).

We applied the same analysis approach to evaluate ABC-IEs in RCC299 [9, 30]. One hundred sixty four ABC-IEs were identified, less than the 221 reported elsewhere [30]. Unlike D-IEs, a single highly conserved motif captured these sequences, all of which were intronic. The IEs further differ from the abundant families in CCMP1545 because they are on average shorter (64 ± 7 nt) than RSIs (152 ± 98 nt), more akin to Introner-Like Elements in fungi [31, 32]. Overall, our results demonstrate that D-IE are an order of magnitude more abundant than repetitive introns reported in genomes of other species, in particular fungi [31–33] and RCC299 [9, 30].

### *Micromonas* proteome comparisons and designation of a new species

**Fig. 4:**
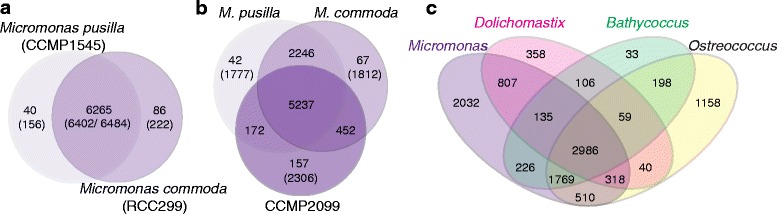
Core, shared, and unique protein-encoding gene families. Values indicate the total number of protein families (containing two or more proteins) and in parentheses the gene count contained within those families for (**a**) *M. commoda* RCC299 and *M. pusilla* CCMP1545. Unique singleton genes are not represented in this panel, and only one representative was kept for identical paralogs in CCMP1545 (30) and RCC299 (21). **b** Comparison of RCC299, CCMP1545, and transcriptome-based CCMP2099 protein sequences. Numbers in parentheses are genes for which no paralog was identified. **c** Comparison of *Micromonas* (combined CCMP1545, RCC299 and CCMP2099), *Dolichomastix tenuilepis*, *Bathycoccus prasinos*, and *Ostreococcus* (combined *O. lucimarinus* and *O.* sp. RCC809). Note that an individual gene family in outer lobes can be composed of two or more predicted proteins from one species

Protein families in the two genome-sequenced *Micromonas* were compared using two approaches. Previous studies have performed global analyses on niche differentiation between the two *Micromonas* [6] and other Mamiellophyceae [18, 19] using best blast approaches. Here, OrthoMCL analysis [34] showed that a total of 6,265 predicted protein families were present in both CCMP1545 and RCC299, encompassing 6,402 and 6,484 proteins, respectively (Fig. 4a). Forty additional families (collectively containing 156 proteins) and 3,337 ‘singleton’ proteins were present in CCMP1545 only. Eighty-six families (together containing 222 proteins) and 3,527 singletons were present in RCC299 only. We minimized the probability of grouping proteins inaccurately (e.g., based only on presence of a common domain) in this analysis by requiring that at least 60 % of the length of the shorter protein overlap with the longer pair member. However, this approach could result in missed orthologs. Therefore, we also used a lenient reciprocal best blastp hit approach (E-value ≤10^−5^), which identified 8,141 putative orthologs. Thus, on average 19 % (reciprocal blastp) to 36 % (OrthoMCL) of predicted proteins can be considered present in one *Micromonas* but not the other, depending on the method used. One gene family expansion in CCMP1545 relative to RCC99 involved SLR proteins. Eighty-four are present in CCMP1545, compared to nine in RCC99. SLR proteins are widespread in eukaryotes and bacteria, typically have low similarity levels, and are involved in a wide range of processes, including cellular stress, regulation of mitosis, and assembly of membrane-bound protein complexes [35]. The significant expansion in *M. pusilla* (CCMP1545) provides an example of expansion that may relate to basic biological differences between these two isolates.

Collectively, the differences observed here and in prior studies [6, 8, 10] clearly support Clade A (RCC299) and Clade D (CCMP1545) as being different species. Therefore, here we name RCC299 *Micromonas commoda* based on molecular diagnoses and the protocols of the International Code of Nomenclature for Algae, Fungi and Plants. The species name refers to the fact that RCC299 is easy to grow in an axenic state in the laboratory. This naming will avoid confusion in the literature [36, 37] where Clade A strains such as RCC299 or its close relatives (e.g., Clade C isolate Mp-Lac38) are incorrectly termed *Micromonas pusilla*.

***Micromonas commoda*** van Baren, Bachy and Worden, **sp. nov**. – Fig. 2b.

*Morphological description* — Naked cells, oblong. Motile with a single flagellum, conserved microtubule arrangement and a flagellar hair of uncharacterized length. The single chloroplast contains a starch granule and pyrenoid. By Coulter Multisizer analysis average diameter (blind to orientation) is 1.43 ± 0.16 μm and volume is 1.532 ± 0.545 μm^3^ during mid-exponential growth (mu = 1.09 d^−1^) under 90 μmol photons m^2^ sec^−1^ (photosynthetically active radiation) at 21 °C in K-medium with an artificial sea water base.

*Molecular description* — Sequences describe the type specimen (RCC299, deposited at the National Center for Marine Algae and Microbiota (NCMA) as CCMP2709) and are available in GenBank under accession numbers KU612123 (ribosomal RNA operon) and XM_002507645 (β-tubulin).

*Molecular diagnosis* — Nucleotide character state “A” in positions 1343, 2455, 2761 and 2795 and “T” in position 2947 of ribosomal RNA operon sequence. These characters are also shared by all Clade A and Clade B *Micromonas* strains (*sensu* Slapeta et al. *MBE* 2006, Simmons et al. 2015) but not *Micromonas pusilla* (Clade D) or Clades C and E (*sensu* Slapeta et al. *MBE* 2006, Simmons et al. 2015). Nucleotide character state “T” in positions 120, 222, 1011 and 1233, and “A” in positions 181 and 1429, and “C” in position 186 of β-tubulin coding sequence. Multiple genes contain a repetitive intron sequence with the motif in Fig. 3C; this sequence is present in closely related lineages (*Micromonas* A/B/C lineage *sensu* Slapeta et al. *MBE* 2006, Simmons et al. 2015) but not in *Micromonas pusilla.*

*Holotype* — Strain CCMP2709 is the type specimen and is preserved in a metabolically inactive state at the NCMA (https://ncma.bigelow.org/). CCMP2709 was deposited at the NCMA by the Worden lab after rendering the field isolate RCC299/NOUM17 clonal and axenic. RCC299 was collected on 10 February 1998 in open ocean surface waters of the South Pacific at 22.3° S, 166.3° E and is available at the Roscoff Culture Collection (http://roscoff-culture-collection.org/).

*Validating illustration* — Figure 2b.

*Habitat and ecology* — Planktonic photosynthetic lifestyle in marine photic zone waters. Habitat extent known to date: coastal to open oceans; has not been observed in high latitude systems (latitudes >60° N or S).

*Etymology* — The specific epithet *commoda* refers to the ‘ease and convenience’ of culturing and propagating this species which grows well in artificial seawater when amended according to K or L1 [38] medium recipes and in other standard marine algal media.

### Comparative genomics of marine green algae

We also compared *Micromonas* with other Class II prasinophytes using predicted proteomes from either genomes or RNA-Seq transcriptome assemblies. First, we compared the proteomes predicted for *M. pusilla* CCMP1545 and *M. commoda* RCC299 to a protein set predicted from the CCMP2099 transcriptome [39, 40], which represents the polar *Micromonas* Clade E2 [9]. After removing duplicates from the transcriptome assembly, a total of 9,494 CCMP2099 proteins were analyzed, ranging from 30 to 7,612 amino acids (average 587 aa). The relative overabundance of short proteins indicates that the transcriptome-based gene assemblies were often incomplete.

OrthoMCL was used to create core, shared and unique protein families between CCMP1545, RCC299, and CCMP2099 (Fig. 4b). ‘Unique’ features in transcriptome-based predicted proteomes can only be determined if the respective proteins are absent from genome-sequenced taxa, but not the reverse: Absence from a transcriptome-based proteome can reflect either absence from the genome, or lack of transcription at the time of sampling. A total of 5,237 families were shared between *Micromonas* Clades A, D and E2 (Fig. 4b). An additional 2,246 families were shared by CCMP1545 and RCC299, possibly reflecting incomplete coverage of the CCMP2099 proteome. CCMP2099 and RCC299 shared 452 gene families not present in CCMP1545, and 172 gene families were exclusive to CCMP1545 and CCMP2099. This suggests CCMP2099 is somewhat less diverged from RCC299 than from CCMP1545, at least in terms of gene content.

CCMP2099 contained putative paralogs absent from CCMP1545 and RCC299. One group contained three genes encoding one or more discoidin domains (i.e., DS or F5/8 type C domains; Pfam 00754). The transcripts from these are divergent, making it unlikely that they represent alternative spliceoforms of a single gene. None of the proteins have predicted transmembrane domains or signal peptides, but the protein predictions do not start with methionine and are likely to be 5′ incomplete. Discoidin proteins are involved in binding of cell-surface attached carbohydrates and are present in eukaryotes and prokaryotes [41]. A recent publication proposed that CCMP2099 is capable of phagotrophy [42], in which case it seems possible that these proteins may function in substrate recognition. Absence of these domains in the other *Micromonas* clades analyzed would then be consistent with the fact that only photoautotrophic growth has been observed for the Clade A and D isolates.

The number of “unique” genes and families in each *Micromonas* clade is extensive and raises questions on similarity levels between orthologs shared between two or more clades. On average, amino acid identity between *Micromonas* orthologs was low, for example when 8,141 reciprocal best blast hits (orthologs) were aligned between CCMP1545 and RCC299, on average only 60 % of amino acid residues were identical over the length of the alignment (Table 3 and Additional file 1: Table S2). Average coverage was 78 %, based on the fraction of aligned regions in the shorter sequence. Results were similar for orthologs shared between CCMP2099 and RCC299 and those shared between CCMP2099 and CCMP1545. In contrast, the 6,449 OrthoMCL-identified orthologs in *O. lucimarinus* and *Ostreococcus* sp. RCC809 averaged 73 % identity and 93 % coverage. Comparison of *O. lucimarinus* and CCMP1545 orthologs (5,534 proteins) showed 54 % identity with 83 % average overlap. This makes ortholog coverage between *O. lucimarinus* and CCMP1545 higher than between RCC299 and CCMP1545. While further investigation is necessary, these results suggest there may be less evolutionary divergence between core genes shared by the most basal *Micromonas* (Clade D, represented by CCMP1545) [9] and its sister group *Ostreococcus* (and *Bathycoccus*), than with the more derived *Micromonas* Clades A and E2.

**Table 3:** Ortholog similarities among prasinophytes with sequenced genomes

Compared organisms	CCMP1545	CCMP1545	RCC299	CCMP1545	*O. lucimarinus*
(pairs)	RCC299	CCMP2099	CCMP2099	*O. lucimarinus*	*O*. RCC809
ID (%)	60	57	57	54	73
Coverage (%)	78	74	77	83	93
Gene fraction (%)	83	62	65	76	88
Ortholog count	8141	5859	6160	5534	6449

The gene fraction represents the ortholog count divided by the total number of predicted proteins in the smaller member of the pair (with respect to gene count)

### Scaled or naked

To understand differentiation between scaled and naked prasinophytes as well as other genus level differences, four additional proteome sets were created and compared. The first three sets comprised (i) all *Micromonas* (CCMP1545, RCC299 and CCMP2099 combined), (ii) all *Ostreococcus* (*O. tauri, O. lucimarinus* and *O.* RCC809) and (iii) the predicted proteome from the *B. prasinos* genome [19]. The fourth set contained just a predicted proteome (transcriptome-based) of the more basal Class II prasinophyte *Dolichomastix tenuilepis* (Fig. 1). Members of the *Dolichomastix* genus are motile [3] and are present in the Arctic and temperate oceans [43]. The four Class II prasinophyte genera shared 2,986 of 10,735 protein families (Fig. 4c). *D. tenuilepis* caused the largest reduction in core numbers and, when excluded, the Class II prasinophyte core is just 9 % smaller than the *Micromonas* core set (Fig. 4b). The *D. tenuilepis* protein set consisted of 16,884 unique proteins, of which 25 % were between 30 and 100 amino acids long (Additional file 2: Figure S2). In contrast, only 2 % of CCMP1545 and RCC299 protein predictions are <100 amino acids, with the smallest being 39 and 33 amino acids, respectively. This suggests issues with the predicted *D. tenuilepis* proteome arising from library construction, RNA sequencing or assembly methods [39]. Alternatively, incomplete protein predictions might have caused issues connected to OrthoMCL criteria on protein overlap. Hence, a conservative estimate of the Class II prasinophyte core excludes *D. tenuilepis*, resulting in 4,755 shared families (44 %).

Both *Bathycoccus* and *Dolichomastix* form scales [44, 45] as do nearly all prasinophytes described to date [46, 47], making their absence in *Micromonas* and *Ostreococcus* unusual. The biosynthetic pathway for scale formation is unknown [48], but four gene families have been reported as expanded in *B. prasinos*, compared to other genome-sequenced Class II prasinophytes: sialyltransferases, sialidases (neuraminidases), ankyrin-repeat proteins, and zinc finger proteins [19]. Here, of 106 gene families shared between *B. prasinos* and *D. tenuilepis* that were not found in the other sequenced genera, 31 were sialyltransferases (Pfam 00777), and 11 were neuraminidases (IPR011040). The sialyltransferase families contained 34 *B. prasinos* and 32 *D. tenuilepis* proteins and the total number in these organisms was even higher, 78 and 71, respectively. Sialyltransferases were otherwise found only in *M. commoda* and *O.* RCC809 (one each), making these genes reasonable candidates for investigation of scale formation.

About half of the 23 *B. prasinos* and 24 *D. tenuilepis* neuraminidases belonged to families shared between the two species, but none were shared with naked prasinophytes. No neuraminidases were found in RCC299, *O. tauri*, and *O.* RCC809, one neuraminidase was present in CCMP1545. *O. lucimarinus* contained four other neuraminidases. Blastp of the *B. prasinos* proteins against the NCBI nr database revealed just single neuraminidase proteins in the Trebouxiophyceae *Chlorella variabilis*, *Coccomyxa subellipsoidea* C-169, *Auxenochlorella protothecoides*, and *Helicosporidium sp*. ATCC 50920. Hence, these proteins provide a second example of enrichment that is potentially related to scale formation.

Another protein present only in *B. prasinos* and *D. tenuilepis* was a Golgi-targeted xyloglucan fucosyltransferase. Xyloglucan is a hemicellulose that makes up ~20 % of the primary cell wall of vascular plants [49]. Other enzymes for xyloglucan synthesis such as xyloglucan endo-transglycosylase/hydrolase (XTH) and β1 → 4-glucan synthase are present in charophyte algae [50], but not in *B. prasinos* or the *D. tenuilepis* transcriptome. *A. thaliana* fucosyltransferase 1 (AtFUT1) has been shown to fucosylate xyloglucan and at least two of the remaining nine AtFUT proteins appear to have some function in cell wall formation [51, 52], suggestive of a possible role of FUT in wall or scale formation in these prasinophytes. In contrast to prior genome-based studies on scale formation [19], we did not find enrichment of ankyrin repeats or zinc fingers in the families shared only between the scaled taxa. Many zinc finger and ankyrin repeat genes were found in *Micromonas* (408 and 132 in CCMP1545, 425 and 129 in RCC299, respectively) and *Ostreococcus* (*O. tauri*: 230 and 69; *O. lucimarinus*: 60 and 75; and *O.* RCC809: 213 and 57). The majority of these were in families present in all the prasinophytes analyzed.

When the Class II prasinophyte proteomes were analyzed together, 19 % (2,032) of the 10,735 protein families identified were exclusive to *Micromonas* (Fig. 4c). This is higher than the fraction of proteins unique to the three *Ostreococcus* (11 %), which represent three of the four *Ostreococcus* clades [53], *B. prasinos* (0.3 %), and *D. tenuilepis* (3 %). For the latter two, inclusion of genomes or transcriptomes from other members of the genus might expand the observed values. These results highlight the greater gene diversification within the *Micromonas* genus compared to *Ostreococcus* and likely more extensive genome reduction prior to divergence within the *Ostreococcus* genus.

Each genome sequenced Class II prasinophyte genus has low redundancy within protein families. Among the families identified here, only 4 % of those that contain CCMP1545 proteins have more than one CCMP1545 protein (Additional file 1: Table S3). The same is true for RCC299, *B. prasinos*, *O. tauri,* and *O. lucimarinus* while *O.* RCC809 shows even less expansion (3 % of families). Low gene family expansion makes these organisms strong candidates for future experimental work on protein function.

### The Viridiplantae ancestor

**Fig. 5:**
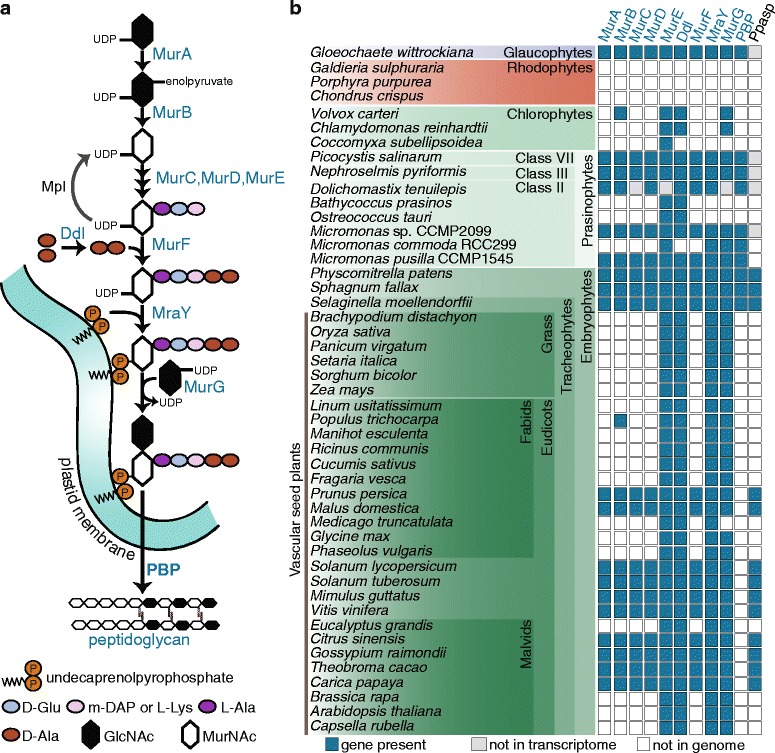
Peptidoglycan biosynthesis. **a** Proteins involved in the peptidoglycan biosynthetic pathway and final crosslinking steps (after [63, 110]). **b** Peptidoglycan pathway proteins in the Archaeplastida, showing MURE, DDL, MRAY and MURG are present throughout the Viridiplantae. The full PG pathway complement with the exception of PBP occurs in several major plant lineages. The final column indicates the gene for plant peptidoglycan associated streptophyte protein (PPASP) identified herein, a putative assignment based on its exclusive presence in plants with the complete or nearly complete PG-pathway

Presence/absence patterns between prasinophyte protein families provide insights into the evolution of the Viridiplantae as a whole. This is true at the level of individual protein families, biosynthesis pathways [40] and the ancestral suite of photosynthesis-related machinery. One such protein is phytochrome, a master regulator in plants that is present in *M. pusilla* and *D. tenuilepis*, but not in *M. commoda*, the *M. sp.* CCMP2099 transcriptome, *Ostreococcus*, *Bathycoccus* or chlorophyte algae. The prasinophyte phytochromes share conserved signaling mechanisms with land plants but detect shorter wavelengths [24]. Here, the ‘unique’ overlap of families containing *Micromonas* representatives and other Class II prasinophytes was greatest with *Dolichomastix* (Fig. 4c; Additional file 1: Table S4). Our analysis identified multiple enzymes involved in peptidoglycan biosynthesis (Fig. 5a) in protein families shared by *D. tenuilepis* and two of three *Micromonas* species (Fig. 5b).

Peptidoglycan (PG) formation involves ten core enzymes, seven of which participate in the conversion of UDP-N-acetyl-D-glucosamine (GlcNAc) to GlcNac-N-acetylmuramyl-pentapeptide-pyrophosphoryl-undecaprenol [54] (Fig. 5a). In bacteria, including cyanobacteria, this compound is transferred to the periplasm by MURG and MRAY, and multiple linear strands are then cross-linked by penicillin-binding proteins (PBPs) to form the 3-dimensional structure of the cell wall PG layer [55, 56]. Glaucophyte algae, which also belong to the Archaeplastida (Fig. 1), maintain the PG-wall of the cyanobacterial endosymbiont around their chloroplast [57]. However, PG has not been observed in plastids of other Archaeplastida groups and is presumed lost, resulting in modifications of the mechanisms for chloroplast division or wall formation that are not understood [57, 58]. In the vascular plant *A. thaliana*, only four PG pathway genes remain: *MURE*, *MRAY*, *MURG*, and *DDL* [58, 59] (Fig. 5b). Indeed, among streptophytes, the complete set of enzymes has only been reported in *P. patens* [58, 60] and *Selaginella moellendorffii* (spike moss), a non-seed species that belongs to the oldest extant vascular plant division [61]. A PG-layer has not been observed in chloroplasts of these taxa.

We identified complete PG pathways in *M. pusilla*, *M. sp.* CCMP2099, and prasinophytes from Class III and VII (Fig. 5b and Additional file 1: Table S5). Most genes are also present in the *D. tenuilepis* transcriptome. In contrast, *M. commoda* has only *MURE*, *MRAY*, *MURG* and *PBP*, and the three *Ostreococcus* as well as *Bathycoccus* only contain *DDL* and *MURE* (Additional file 1: Table S5). Two of these enzymes (*MURG* and *PBP*) were reported previously in RCC299 (*M. commoda*) [59]*.* Chlorophyte algae also have only a few PG-pathway genes and show differences between *Coccomyxa subellipsoidea* versus *C. reinhardtii* and *Volvox carteri* (Fig. 5b, Additional file 1: Table S5). A PG layer has not been observed around the chloroplast of *Micromonas* (or elsewhere, Fig. 2a, b) or other prasinophytes, at least by conventional transmission electron microscopy imaging. The gene patterns observed here with expanded taxon sampling illustrate differences in the PG-pathway ranging from complete retention (e.g., *M. pusilla*, Class III and VII prasinophytes) to differential PG-pathway losses between some *Micromonas* species, other Class II prasinophytes and chlorophyte algae (Fig. 5b). These findings led us to investigate PG biosynthesis in a broader sampling of Archaeplastida lineages.

We characterized PG pathway gene complements in the Viridiplantae by searching protein families in the Phytozome database, version 11 [62]. The mosses *Physcomitrella patens*, *Sphagnum fallax*, and *Selaginella moellendorffii* contained the complete pathway, including PBP, as expected based on prior literature on *P. patens* [60, 63]. Each gene in the pathway was represented by a single Phytozome gene family, except PBP, which was not represented (see methods; Additional file 1: Table S5). Surprisingly, we also identified a full complement of PG pathway genes except PBP in taxa distributed throughout the streptophytes (Fig. 5b, Additional file 1: Tables S5 and S6). Most of these protein sequences are derived from automated gene predictions, therefore we verified expression using the predicted protein sequence as a tblastn query against EST databases (Additional file 1: Table S6). Evidence for expression was found for all genes, although not necessarily from all species (possibly because available EST data in GenBank is sparse for many streptophytes). Representative orthologs of each PG pathway protein have predicted chloroplast transit peptides (Additional file 1: Table S7) and AtMURE has been shown to localize to the plastid, where it is thought to function in chloroplast biogenesis [64].

For the vascular seed plants that did not contain the full pathway (including *A. thaliana*, see e.g., Fig. 5b), four genes were usually present: *MURE* (Phytozome gene family #63996886), *MRAY* (#63772898) and *MURG* (#63987572), as well as *DDL* (#63770242). This four-gene subset encodes enzymes that function in the second half of the PG-biosynthesis pathway. Interestingly, DDL creates the D-alanyl-D-alanine dipeptide that is linked to the MurNac tripeptide by MURF (Fig. 5a), but *MURF* itself is not part of the subset, suggesting an as yet unrecognized functional equivalent may exist.

Unlike the patchiness seen across the Viridiplantae, none of the relevant enzymes are harbored in available red algal genomes, while all are present in the glaucophyte *Gloeochaete wittrockiana* (Fig. 5b, Additional file 1: Table S5). The latter finding corresponds well with the detection of a PG-wall in glaucophyte chloroplasts as well as several enzymes involved in the PG-pathway [57]. Collectively, our results indicate that members of several prasinophyte classes represent a more ancestral Archaeplastidal state than a variety of land plants, the more reduced Class II prasinophytes (*Ostreococcus* and *Bathycoccus*) and chlorophyte algae. Moreover, rather than a very limited presence within the Viridiplantae [61], the majority of the PG pathway has been retained throughout plant evolution and selective losses have occurred in multiple independent events (Fig. 5b).

**Fig. 6:**
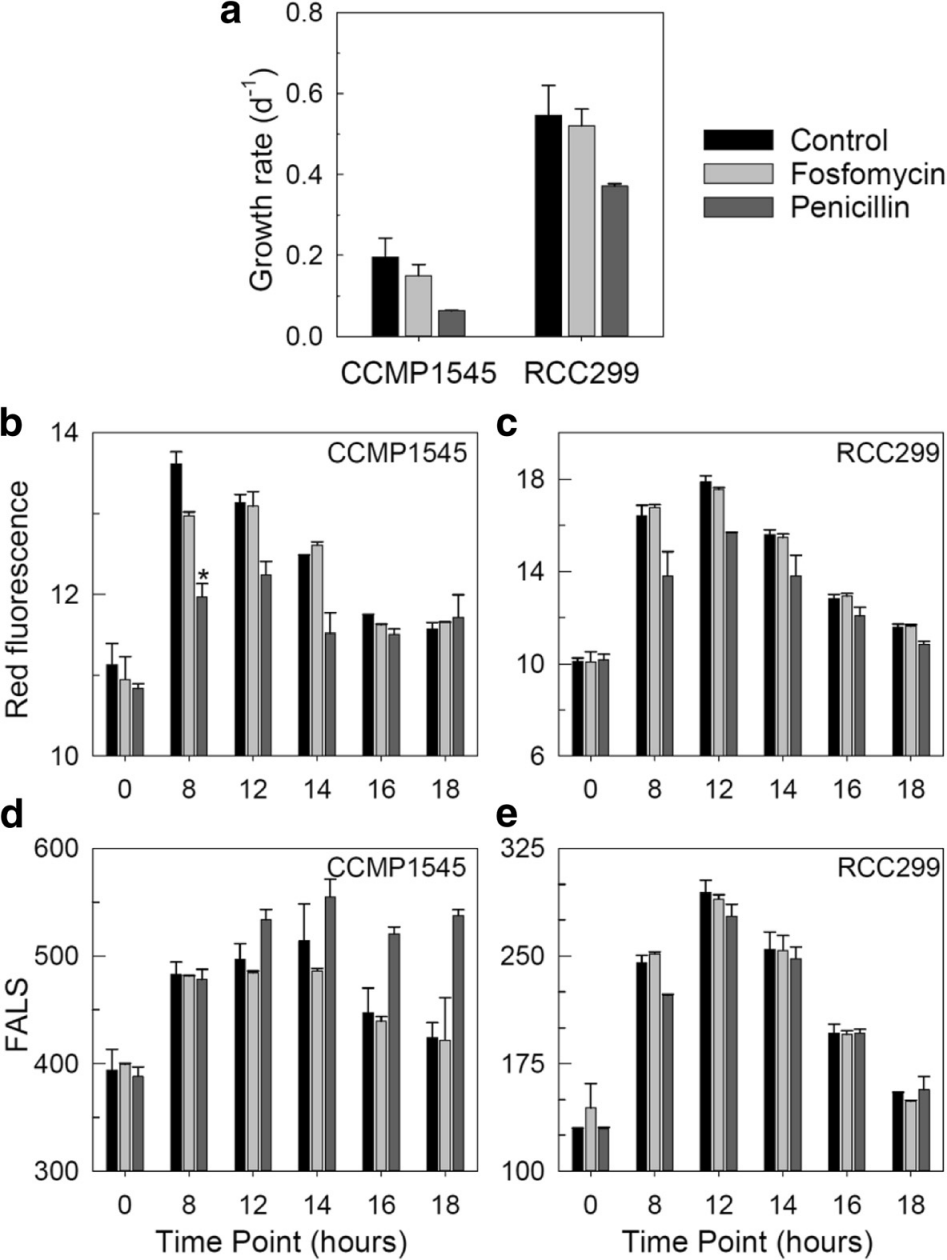
Antibiotic treatments in exploratory experiments on *M. pusilla* and *M. commoda*. Treatments with 10 mM fosfomycin and penicillin show changes between the penicillin treatment and control at each time point but, apart from the 8 h time point (* indicates *p* < 0.005), these differences are not significant (*p* > 0.05). Antibiotics were added 1 h after lights on (0 h timepoint) to cultures acclimated to a 14 h light/10 h dark cycle. Thus, 14 h was 1 h after lights off and the 18 h timepoint was 5 h into the dark period. **a** Growth rate of CCMP1545 and RCC299 in controls and treatments. Chlorophyll-derived red fluorescence in **b** CCMP1545 and (**c**) RCC299 normalized to bead standards. Forward angle light scatter (FALS), an indicator of cell size in (**d**) CCMP1545 and (**e**) RCC299 normalized to bead standards. Error bars reflect the standard deviation of biological duplicates

Penicillin inhibits PBP activity in bacteria and fosfomycin acts earlier in the PG-pathway, on MURA [65, 66]. We performed exploratory experiments on *Micromonas* using these antibiotics. *M. pusilla* grew slower than *M. commoda* in our experiments (Fig. 6a). Controls followed the expected [24] increase in chlorophyll-derived red fluorescence (RED) and forward angle light scatter (FALS, an indicator of cell size) that occurs during the light period, and their reduction as division begins at night fall (Fig. 6b-e). The only significant change from controls occurred at the 8 h time point in *M. pusilla*, where RED was lower (*p* < 0.005) in the penicillin treatment than the control and this reduction appeared to persist in subsequent timepoints. Other differences were not significant (*p* > 0.05) in part due to the low power of duplicates in statistical analyses. Nevertheless, general trends throughout the time course were identified. Cell division appeared to cease in the *M. pusilla* penicillin treatment based on the growth rate (Fig. 6a) and, independently, the lack of reduction in FALS (that normally occurs with cell division; Fig. 6d). These results are suggestive of an active role for PBP in *M. pusilla* and are consistent with experiments on other Archaeplastida that contain PBP. In glaucophytes including *Gloeochaete*, antibiotics cause cessation of chloroplast division and loss of pigments (which would cause a reduction in red fluorescence) [67]. In *P. patens* antibiotic treatment results in fewer chloroplasts and development of macrochloroplasts [68]. In knockout experiments on *P. patens MURE* and *PBP*, a macrochloroplast phenotype is observed in the former and inhibition of division in the latter [58]. Thus, the PG-pathway has been proposed to function in chloroplast division in glaucophytes and moss. Additional experiments with greater replication as well as visualization with chemical-fixation free electron microscopy are important next steps to test our hypothesis that the PG-pathway has similar roles in multiple prasinophyte lineages.

We also investigated whether plant species with the complete PG biosynthesis pathway (excepting the cross-linker PBP, Fig. 5b) share other genes that are absent from the reduced PG-pathway taxa. To this end, we searched Phytozome for shared families that are absent from organisms with just the 4-gene PG subset. Phytozome gene family #63760547 matched these criteria, all but one of its 18 members (13 organisms) contain a C-terminal LysM domain (Pfam 01476). Seventeen members also have a conserved N-terminal 21 amino acid domain (Additional file 2: Figure S3) that is almost entirely hydrophobic and is predicted to form an alpha-helical transmembrane region, with the LysM domain on the outside. In bacteria, the LysM domain binds peptidoglycan [69]. In plants, LysM domain-containing proteins are thought to be involved in sensing bacterial peptidoglycan [70] and chitin [71] by recognizing N-acetylglucosamine moieties [69]. When we performed additional motif searches against Phytozome using the transmembrane and LysM motifs together, we found the bi-domain protein in all embryophytes that contained the PG synthesis pathway, regardless of PBP status (Additional file 1: Tables S5 and S6). Most of these taxa have one or two orthologs and the mosses *Sphagnum fallax* and *P. patens* have three. We named this bi-domain protein “Peptidoglycan Pathway Associated Streptophyte Protein (PPASP)” because it was not detected in the NCBI nr database (except in streptophytes with the nearly complete pathway) or in the glaucophyte and prasinophytes studied here. Phylogenetic analyses of MURE (as a representative of the four-gene subset), MURA and PPASP showed branching patterns that follow known relationships among plants (Additional file 2: Figure S4). The presence/absence patterns of PBP and PPASP across the Archaeplastida suggest that PPASP evolved or was acquired around the time that streptophytes diverged from the ancestor shared with chlorophyte and prasinophyte algae. PPASP would then have been differentially lost in taxa from multiple plant lineages, alongside other PG-pathway components.

The discovery of several vascular plant lineages containing most of the PG-pathway, and presence of the shared four-gene PG-subset in others, is suggestive of selective retention and implies a cellular function. The proteins necessary for transmembrane transport and synthesis of the lipid intermediates (MRAY and MURG) are present in all plants. We speculate that plants with the full PG pathway (excepting PBP) synthesize the PG lipid intermediate, which is then localized between the inner and outer chloroplast membranes. PPASP does not appear to contain a transit peptide, but could be inserted into the outer chloroplast membrane to interact with the N-acetylglucosamine moieties of the pentapeptide (see Fig. 5a). If the PPASP innovation in terrestrial plants is connected to the PG-pathway it may serve as an alternative modality to PG-formation (and PBP activity) by incorporating pathway intermediates into as yet uncharacterized components of the plastid wall. Our findings raise new questions regarding the PG-pathway role in extant plants and prasinophytes by highlighting complexity in retention of cyanobacterial machinery that likely influences chloroplast division or wall structure.

### The core of photosynthesis

One approach to understanding photosynthesis and plant evolution has been identification of nucleus-encoded proteins shared across the Viridiplantae (Fig. 1) and other photosynthetic lineages but absent from non-photosynthetic organisms. To this end, the “GreenCut” [72] and subsequently the “GreenCut2” [73] were created, the latter using genomes from 20 taxa including diatoms, a red alga, chlorophyte algae (e.g., *C. reinhardtii*), prasinophyte algae (i.e., three *Ostreococcus*), moss, spike moss and several seed plants. The GreenCut2 provides the results as 597 *C. reinhardtii* proteins and their 710 orthologs in *A. thaliana* (677 unique genes). *Micromonas* was not used for generation of the GreenCut2, but a survey was performed using the *A. thaliana* GreenCut2 orthologs against CCMP1545 and the authors concluded that ~10 % (61 in total) of GreenCut2 proteins were missing from *M. pusilla* [73].

**Fig. 7:**
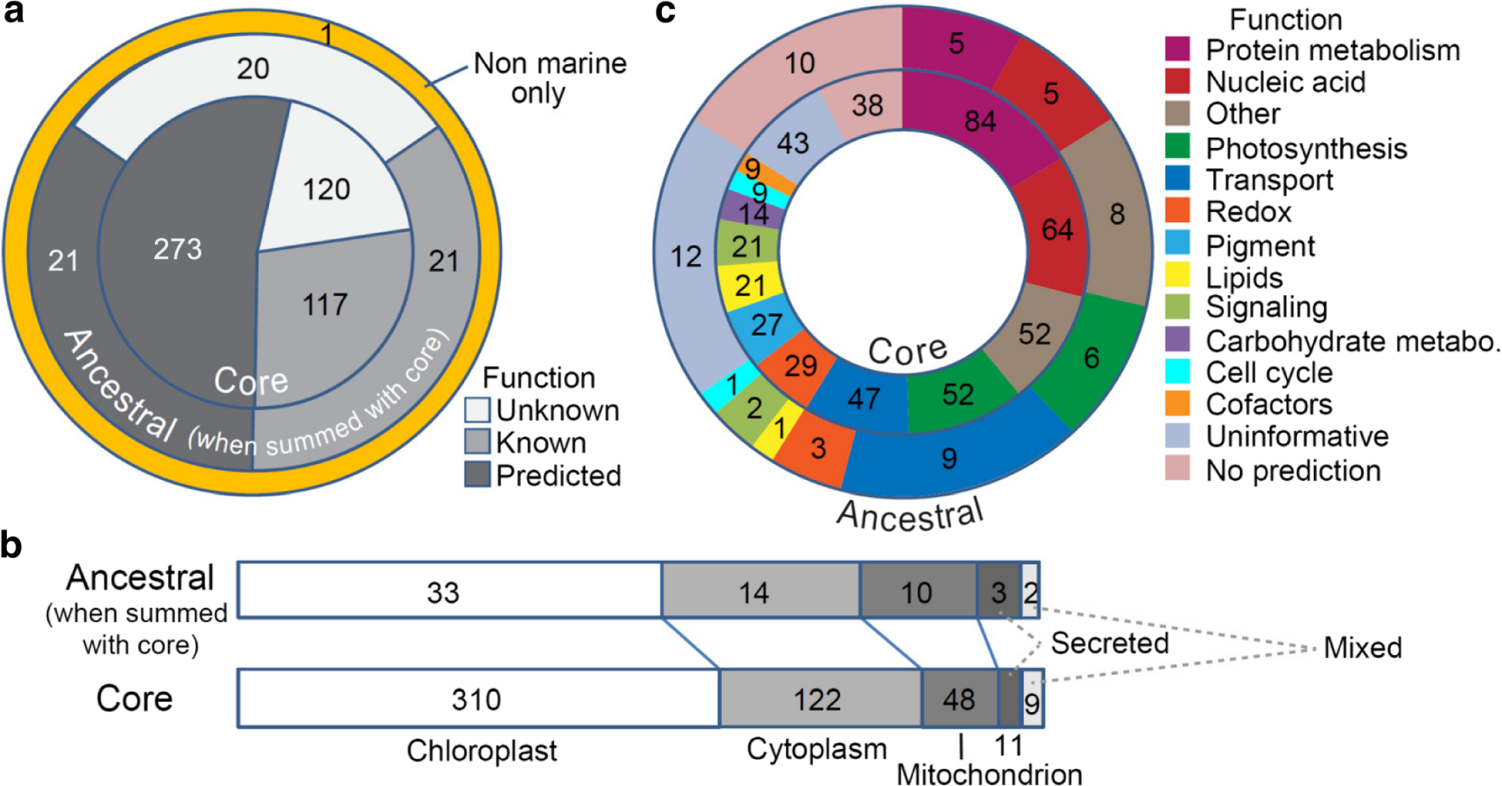
The GreenCut can be divided in ancestral, core and land specific sets. **a** Proteins with unknown functions (white), known functions (light grey) and predicted functions (dark grey) are depicted based on [73]. ‘Other’ refers to a domain or motif suggesting a general function but not a specific functional category. **b** Cellular location of *A. thaliana* protein families in the Ancestral and Core (essential) sets based on TargetP analysis. Mixed: members of an ortholog group show different targeting signals. **c** Functional categories of core and ancestral genes [73], showing that proteins involved in pigment synthesis, carbohydrate metabolism, and cofactors are all maintained in the core set and do not appear to have undergone differential losses

We used OrthoMCL to group *C. reinhardtii* and *A. thaliana* GreenCut2 proteins. This resulted in 573 families containing 591 and 673 of their proteins, respectively. Four reported GreenCut2 proteins (MPA14, two copies of BGS4, and CGL155) were removed because they did not cluster using our criteria of 30 % overlap (of the shorter protein in a pair) and blastp E-value ≤10^−15^. CGL155 has been identified as a potential false positive previously [73]. Of 573 GreenCut2 families, 564 (98.4 %) and 559 (97.6 %) were detected in *M. commoda* and *M. pusilla* respectively (Additional file 1: Table S8). Of those missing, none were in existing chloroplast genome data and five were absent from both genome-sequenced *Micromonas* species. However, one of these five was present in *M. sp.* CCMP2099, two in *D. tenuilepis*, and the other two in *Ostreococcu*s (Additional file 1: Table S8). *O. lucimarinus* and *O.* RCC809 protein sets contained 555 and 547 GreenCut2 families, respectively, and 544 were present in *B. prasinos*. These losses were proposed to represent specialization to life in the marine environment [73]. However, the *Ostreococcus* and *Bathycoccus* genomes are also known to be highly reduced. Our collective analyses show that 99.8 % of our GreenCut2 proteins (after removal of false positives identified above) are present in marine prasinophytes. A single family (one protein; 0.2 %) emerged from the GreenCut2 set as being unique to terrestrial green lineage organisms, a putative nickel/cobalt transporter present in chlorophytes and land plants (Fig. 7a).

The core set of nucleus encoded proteins present in prasinophytes, chlorophytes and streptophytes (but absent from non-photosynthetic taxa) gives insight into the essence of photosynthesis in the Viridiplantae, its control by the eukaryotic host that endosymbiosed a cyanobacterium and the composition of the green algal progenitor. Therefore, we created an Ancestral GreenCut2 (AGreenCut2) that is based on GreenCut2 proteins found in any prasinophyte (as opposed to all) as well as in chlorophytes and streptophytes (as identified in [73]) (Fig. 7, Additional file 1: Table S8). Proteins differentially ‘missing’ from prasinophytes provide information on features that may relate to adaptation to life on land or merely the extreme genome reduction in *Ostreococcus* and *Bathycoccus*. Indeed, a protein set essential to photosynthetic processes should have orthologs in all relevant taxa. We identified these proteins by removing any families that were missing in one or more genome-sequenced taxa and termed them the “Core GreenCut2” (CGreenCut2). Signal peptides targeting the chloroplast are predicted on the majority of *A. thaliana* proteins in the Ancestral (53 %) and Core (61 %) protein families (Fig. 7b). The *M. pusilla* and *M. commoda* ortholog groups show 48 % (Ancestral) and 53 % (Core) chloroplast targeting for these sets. The percentage of *Micromonas* proteins with consistent TargetP-based localization predictions was lower than for *Arabidopsis*, resulting in the lower percentages assigned to the chloroplast-targeted category (Fig. 7b, for *Micromonas* see Additional file 2: Figure S5). Among the Core set were all CGreenCut2 proteins with assigned functions in pigment, carbohydrate metabolism and cofactor synthesis (Fig. 7c). These results emphasize that a more reduced set comprising 510 CGreenCut2 protein families (nucleus-encoded) are critical to photosynthesis in the green lineage.

## Conclusions

The plant lineage as a whole contributes hugely to the uptake of atmospheric CO_2_ and food resources in the ocean and land. Our studies reveal a high level of diversity in Class II prasinophytes. *Micromonas pusilla* (represented by CCMP1545 and other Clade D strains) and *Micromonas commoda* (represented by RCC299, Clade A) have large differences in their genomic organization, each having repetitive intron families (IE) not present in the other [6, 9, 30], and CCMP1545 alone having a high fraction of GC splice donors. Protein families are often not shared between these and/or with other Class II prasinophytes such as the polar *Micromonas* species and particularly *Ostreococcus* and *Bathycoccus*, the more diminutive genome-sequenced genera. Our comparative genome analyses provide candidate genes for future studies on structural and niche defining aspects of these taxa. Perhaps most surprising is that two of the three *Micromonas* species analyzed, as well as *D. tenuilepis*, have the complete peptidoglycan biosynthesis pathway, including the penicillin binding protein responsible for the final crosslinking step. Likewise, in multiple land plants and other prasinophyte algae this pathway is maintained nearly in its entirety (as characterized in bacteria), although previously known only in *P. patens* and largely lacking in *A. thaliana* and other plants. Together with the GreenCut2, AGreenCut2 and CGreenCut2 protein sets, our evidence-based comparative analyses provide a springboard for investigation of key aspects of photosynthesis, requirements for plant adaptation to a terrestrial environment and adaptive processes in marine green algae.

## Methods

### CCMP1545 genome sequence improvement

At the time of initial publication the CCMP1545 genome sequence had ~215 kb of unknown sequence. To perform genome improvement, the CCMP1545 whole genome shotgun assembly was broken down into scaffolds and each scaffold piece reassembled with phrap. These scaffold pieces were then analyzed for GC content and the four scaffolds with the lowest GC content (scaffolds 2, 3, 18 and 19) were selected for genome improvement using a Phred/Phrap/Consed-based pipeline [74]. Initially all low quality regions and gaps were targeted with computationally selected sequencing reactions completed with 4:1 BigDye terminator: dGTP chemistry (Applied Biosystems, Foster City, CA). These automated rounds included walking on plasmid subclones using custom primers. Following completion of the automated rounds, a trained finisher manually inspected each assembly. Further reactions were then manually selected to improve the genome. These reactions included additional custom primer walks on plasmid subclones and fosmids. Smaller repeats in the sequence were resolved by transposon-hopping 8 kb plasmid clones. Fosmid clones were shotgun sequenced and finished to fill large gaps and resolve larger repeats.

Each assembly was validated by an independent quality assessment. This examination included a visual examination of subclone paired ends and visual inspection of high quality discrepancies and all remaining low quality areas. The four improved scaffolds are telomere to telomere, consist of 4,888,335 base pairs of improved sequence with no gaps and estimated error rate of less than 1 error in 100,000 base pairs.

### Culturing for transcriptomics

Axenic clonal derivatives of *Micromonas sp.* RCC299 (deposited at the National Center for Marine Algae and Microbiota, NCMA, as CCMP2709) and *M. pusilla* CCMP1545 were grown in sterile polystyrene culture flasks (BD Biosciences, Franklin Lakes, NJ) in artificial seawater (see http://www.mbari.org/resources-worden-lab/) amended with K (RCC299) [75] or L1 (CCMP1545) [76] media nutrients. Cells were grown at 21 °C under 220 μEinsteins m^−2^ s^−1^ photosynthetically active radiation (PAR) on a 14 h/10 h light/dark cycle. Cell concentrations, side-angle light scatter (SSC) and chlorophyll fluorescence (Red) were monitored using a Beckman Coulter Epics XL4 or BD Accuri C6 Flow Cytometer (BD Biosciences, San Jose, CA) flow cytometer. Fluorescent polystyrene beads (Polysciences, Inc., Warrington, PA) were used as a standard for instrument performance and to assess day to day variability as well as normalization of cellular characteristics (SSC and Red). Cultures were maintained in mid-exponential growth for at least 10 generations. Cells were harvested for RNA 8 h (RCC299) or 5 h (CCMP1545) after the onset of light.

*Dolichomastix tenuilepis* CCMP3274 and *Micromonas sp.* CCMP2099 cultures were obtained from the NCMA (West Boothbay Harbor ME, USA) and grown as previously described [24]. Briefly, CCMP2099 growth was monitored using flow cytometry as above while CCMP3274 growth was monitored by fluorometry (TD-700, Turner Designs, Sunnyvale, CA, USA). After at least five transfers in mid-exponential growth phase cells were harvested for RNA two hours before and after the onset of light.

### Electron microscopy

Cells were grown as above, fixed using 1 % EM Grade glutaraldehyde for 1 h, and further processed according to methods outlined in [77]. Ultrathin (80 nm) sections were cut from the resulting epoxy blocks using an ultramicrotome (Leica) and mounted on 200 mesh copper grids (Ted Pella Inc). Sections were stained for 5 min with 2 % uranyl acetate (aqueous) and 1 min in Sato lead [78]. Sections were imaged using a JEOL 1200 transmission electron microscope operating at 80 kV.

### Proteomics

Proteins from harvested cells (1.6 × 10^8^ – 2.7 × 10^8^) were extracted from whole cell (global), soluble, and insoluble lysate fractions according to established protocols [79–81], with modifications. Briefly, global and soluble fraction proteins were denatured using 8 M urea (final concentration), reduced with fresh dithiotreitol (DTT) at 5 mM (final concentration) and incubated at 60 °C for 30 min. Proteins in the insoluble fraction were suspended in 50 mM NH_4_HCO_3_ (pH 7.8) containing 8 M urea and 1 % CHAPS, fresh DTT solution was added to a final concentration of 5 mM, and incubated as above. Following incubation, an alkylation step was performed by adding a volume of 0.5 M iodoacetamide (IAM) to each fraction (40 mM, final concentration) with an additional incubation for 1 h at 37 °C in the dark. Sequencing-grade trypsin (Roche, Indianapolis, IN) was used for digestion at 1:50 unit-to-protein, and peptides desalted using a strong cation-exchange (SCX), C-18 SPE column (Supelco, St. Louis, MO) following established protocols [80]. Extract protein and peptide concentrations were determined using a BCA assay (Pierce Chemical Co., Rockfort, IL). Two chromatography approaches were used to maximize peptide separation and proteome coverage. First, peptides from the global, soluble, and insoluble lysis fractions were further fractionated off-line (LC not coupled to the mass spectrometer) using reversed-phase, high pH chromatography as previously described [82]. Secondly, a two dimensional on-line (coupled) LC separation was used where the first dimension consisted of a SCX column and the second dimension consisted of a reversed-phase separation column. Additional details concerning this 2D on-line chromatography approach have been previously published [83, 84]. All columns were manufactured in-house by slurry packing media into fused silica (Polymicro Technologies Inc., Phoenix, AZ) using a 0.5-cm sol–gel frit for media retention [85].

Tandem mass spectra were generated using both ThermoFinnigan LTQ and LTQ Orbitrap Velos mass spectrometers (Thermo Scientific, San Jose, CA) according to established protocols [86]. For both instruments, separated peptides were ionized (positive) using an electrospray ionization interface (manufactured in-house; no sheath gas or make-up liquid was used) that consisted of chemically etched electrospray emitters [87] (150 mm o.d. 20 mm i.d). Mass spectrometers were operated using a heated capillary temperature and spray voltage of 200 °C and 2.2 kV, respectively. Data was acquired for 100 min (~1500 min for the coupled 2D-LC), beginning ~60 min after sample injection (~10 min into gradient). Mass spectra (AGC 1x10^6^) were collected from 400–2000 m/z at a resolution of 100 k followed by data dependent ion trap MS/MS spectra (AGC 3x10^4^) of the six most abundant ions (ten most abundant ions for LTQ Orbitrap Velos) using a collision energy of 35 %. A dynamic exclusion time of 60 s was used to discriminate against previously analyzed ions (A dynamic exclusion time of 180 s was used for the LTQ Orbitrap Velos).

Mass spectrometric data were subjected to sequence analysis using the SEQUEST algorithm ([88], ThermoElectron, San Jose, Ca. version 27 (rev. 12)) which compares MS/MS spectra to a sequence repository, specifically the collection of all gene models derived from the genome in [6], described elsewhere. Briefly, SEQUEST input files were created using in-house parent isotope correction algorithm DeconMSn ([89], omics.pnl.gov), a parent mass tolerance of ±3 Da was employed to capture remaining de-isotoping errors, a static modification was applied to all Cysteine residues to reflect alkylation with iodoacetamide (+57.0215 Da), and no proteolytic enzyme was specified. The output from this analysis was then rescored using the MSGF spectral probability algorithm [90]. A subset of the full dataset was analyzed using a target-decoy approach, whereby all sequences are combined with their reverse complement in a single search file, allowing false discovery rates (FDR) to be assessed at given quality level cutoffs [91]. It was observed that an MSGF spectral probability E-value ≤ 10^−10^ provided an FDR of <1 %, and this value was used to filter subsequent data.

### RNA extraction, sequencing and transcriptome assembly

Cultures were filtered onto 0.8 μm Supor filters (PALL Life Sciences, Ann Arbor, MI) under −5 mmHg pressure. Filters were placed in sterile bead beating tubes, flash frozen in liquid N_2_ and stored at −80 °C. RNA was extracted using the TotallyRNA kit (Life Technologies, Grand Island NY, USA). Initially, ~200 μl of autoclaved glass beads (Biospec Products, Bartlesville OK, USA) and 1 ml lysis buffer from the kit were added to the cell pellet or filter. Samples were then bead beat for 1 min. The rest of the extraction followed the manufacturer’s instructions. Genomic DNA contamination was removed using the TurboDNA-free kit (Life Technologies) following manufacturer’s instructions. RNA integrity was evaluated on a bioanalyzer (Agilent, Santa Clara CA, USA) and quantity determined on a QuBit (Life Technologies).

For CCMP1545 and RCC299 RNA-Seq, polyA RNA was isolated from 5 μg total RNA using the Dynabeads mRNA isolation kit (Invitrogen). The isolation procedure was repeated to ensure the sample was depleted of rRNA. Purified RNA was then fragmented using RNA Fragmentation Reagents (Ambion) at 70 °C for 3 min, targeting fragments ranging from 200 to 300 bp. Fragmented RNA was then purified using Ampure XP beads (Agencourt). Reverse transcription was performed using SuperScript II Reverse Transcription (Invitrogen) with an initial annealing of random hexamer (Fermentas) at 65 °C for 5 min, followed by an incubation of 42 °C for 50 min and an inactivation step at 70 °C for 10 min. CDNA was then purified with Ampure XP beads, followed by second strand synthesis using dNTP mix with dTTP replaced by dUTP. Reactions were performed at 16 °C for 1 h. Double stranded cDNA fragments were purified and selected for targeted fragments (200–300 bp) using Ampure XP beads. The ds cDNA were then blunt-ended, A-tailed, and ligated with library adaptors using the Kapa Library Amplification Kit (Kapa Biosystems). Adaptor-ligated DNA was purified using Ampure XP beads. Digestion of dUTP was performed using AmpErase UNG (Applied Biosystems) to remove second strand cDNA. Digested cDNA was again cleaned up with Ampure XP beads, followed by amplification by 10 cycles PCR using the Kapa Library Amplification Kit (Kapa Biosystems). The final library was cleaned with Ampure XP beads. Sequencing was done on the Illumina HighSeq platform generating 326 M paired end reads of 150 bp each.

Library construction and sequencing were different for CCMP2099 and *D. tenuilepis* and also included an assembly step, as described [24]. Like the libraries generated for CCMP1545 and RCC299 a poly-A selection step was employed. We used all predicted proteins as blastp queries against the NCBI nr database and against a database of translated prasinophyte transcriptomes [39] in order to identify potential contamination in the assemblies. CCMP2099 had no hits to bacteria that were superior to those to prasinophytes, and of 10,965 proteins unique to *D. tenuilepis*, 132 had best blast hits (E-value cutoff 10^−10^) with non-photosynthetic bacteria suggesting the bulk of data comes from the alga rather than potential bacterial contaminants in the culture. Likewise, only one full-length 18S rRNA gene sequence each was present in the CCMP2099 and the *D. tenuilepis* transcriptomes (and these matched the published 18S sequences) as were single variants of cytochrome c and actin in each. This indicates that only sequences from the target organism were present in the assembled transcripts.

### CCMP1545 and RCC299 short read alignment and processing

The 326 M paired-end reads generated for each strain were randomly subsampled to 21,751,585 and 22,372,188 paired-end reads and aligned to the CCMP1545v3 and RCC299v3 genome assemblies, respectively. This was performed using Tophat version 1.4.0 [25] with parameters -r 27 --mate-std-dev 100 --max-intron-length 25000 --min-intron-length 20 --solexa1.3-quals --library-type fr-firststrand. In CCMP1545, 16,549 splice junctions were confirmed by more than 10 reads and 10,999 were confirmed in RCC299.

The Tophat associated program Cufflinks [92] merges blocks of overlapping reads into transfrags, but we found that the algorithm tends to concatenate genes when they are closely spaced in the genome. Instead, we used Tophat’s intensity bed track output to determine which strand was most transcribed. Nucleotides with less than 10 covering reads were considered not transcribed. Neighboring transcribed loci (transfrags) were merged into spliced transfrags if a Tophat junction existed between them on the same strand. Alternative splicing occurs, but is not common in *Micromonas*, making these collections of 14,084 (CCMP1545) and 12,659 (RCC299) spliced and unspliced transfrags a reasonable approximation of the transcriptome. Genome-free transcriptomes were also generated using Trinity [93] (version trinityrnaseq_r2012-06-08), with settings --seqType fq --SS_lib_type RF --CPU 10 --JM 50G. This program also has trouble with densely populated genomes, in this case leading to a fragmented transcriptome. The resulting assemblies (51,118 for CCMP1545, 47,719 for RCC299) were aligned to the respective genomes using Blat [94]. Despite the fragmentation, Trinity transcripts often aligned across gaps in the CCMP1545 genome, filling in missing nucleotides. Seventeen genes in the final CCMP1545 gene set were based on Trinity transfrags.

To determine whether a predicted gene was expressed, we used Cufflinks with the parameters described above, but added the gene models using the -G parameter. Any transcripts with FPKM < 2000 (out of a total range between 0 and 5.8e + 8) were considered not transcribed.

### Evidence-based modeling of the CCMP1545 and RCC299 gene sets

JGI allgenes sets are non-redundant combinations of gene models created by several gene predictors and can be downloaded from http://genome.jgi.doe.gov/. These gene models were 5′ and 3′ extended where possible by the transfrags, and ORFs were repositioned accordingly. These extended gene models then went through a second round of extension and ORF prediction.

All gene models were then scored based on intron evidence (+20 if all introns in a gene model were confirmed by ESTs or Tophat junctions, +5 if some introns were confirmed, −5 if none were, +20 for every intron that overlapped an introner element), peptide evidence (+1 for every MS peptide found in the gene model ORF if the peptide could only be mapped to one genomic location, otherwise +1/number of locations), previous manual annotation (+10000), full cDNA overlap (+20 if both the forward and reverse ESTs of a cDNA overlapped the gene model) and completeness (+5 per UTR if EST or transfrag derived UTRs were present in the gene model). Overlapping gene models were then grouped into loci, and for every locus the highest scoring combination(s) of non-overlapping genes was selected.

Gene merges and breaks still occurred in this highest scoring gene set, therefore we created tracks for a local installation of the UCSC genome browser [95] to display all gene models and the transfrag, junction, peptide and EST evidence and visually scanned the whole genome to confirm and correct the gene set. At this stage, Trinity transcripts were used to merge genes across gaps and sometimes to infer the missing nucleotide and amino acid sequences.

### Introner element identification

CCMP1545 directional Sanger sequenced ESTs (available at http://genome.jgi.doe.gov/MicpuC3/MicpuC3.download.html) were aligned to the genome using Blat. Of 28,686 ESTs, 17,708 were single exon. Multi-exon ESTs were oriented based on their splice donor and acceptor sequences (G[TC]/AG and AT/AN). Eight hundred twenty-six ESTs for which the transcribed strand could not be determined were discarded. Of the remaining 10,978 ESTs, nonredundant introns of 500 nt or shorter were selected (4,403 introns) and their (reading strand) sequences clustered using Blastclust version 2.2.21 (available through http://www.ncbi.nlm.nih.gov/IEB/ToolBox) with settings- S 80 -L 0.80 -p F. This resulted in three groups of 966, 72, and 66 introns, respectively. All three groups were subjected to the meme motif finder [96] (the meme suite version 4.6.1) with parameters -dna -mod zoops -nmotifs 6 -minsites 10 -maxsize 200000. Introner element positions were determined using fimo [97] (part of the meme suite) at --output-pthresh 1e-10 with the seven selected meme motifs on the CCMP1545 genome. The motifs detect different parts of the introner elements, therefore hits were concatenated if they occurred within 50 nt of each other. Splice donor consensus sequences and logos for IE containing and non-IE containing introns were also created using meme on a random subsample of 500 sequences for each group using the fasta-subsample script in the meme suite. Meme parameters were -dna -mod oops -nmotifs 1 -w 12. For IE identification in RCC299 predicted introns (from gene models described in this paper) were clustered as described for CCMP1545, resulting in a single group of 41 introns. Meme identified a 50 nucleotide motif that was subsequently used on the RCC299 genome sequence to identify 164 ABC-IE.

### Protein clustering and gene family assignment

Refseq protein sets for *O. tauri* and *O. lucimarinus*, were downloaded from Genbank. *O. RCC809* proteins were obtained from JGI (http://genome.jgi.doe.gov/OstRCC809_2/OstRCC809_2.download.html) and *B. prasinos* Bban7 proteins from https://bioinformatics.psb.ugent.be/gdb/bathycoccus/RELEASE_15jul2011. *M. sp.* CCMP2099 and *D. tenuilepis* proteins were based on translation of short read contigs [24] using ESTScan v3.0.3 [98] with default settings and a training set consisting of all RefSeq mRNA entries under the Bacillariophyta classification. Peptides shorter than 30 aa were removed from the results.

For creating multiple species ortholog sets, we used OrthoMCL [34] according to the user manual, with the E-value cutoff set to 10^−15^. PercentMatchCutoff was set to 60 in the comparison of RCC299 and CCMP1545. Duplicate genes (30 in CCMP1545 and 21 in RCC299) were removed before analysis. For all other OrthoMCL analyses percentMatchCutoff was set to 30 to allow for the greater evolutionary distance between organisms. The same OrthoMCL parameters were used to create GreenCut2 families from proteins that were downloaded from JGI and TAIR by following URLs for every gene listed in Supplemental File 2 of [73]. To find prasinophyte orthologs, the individual GreenCut2 proteins were used for reciprocal best blastp (E-value cutoff 10^−5^) against prasinophyte protein sets. A GreenCut2 gene family was counted as present when at least one of the family members had a reciprocal best hit with a protein from the species under consideration. The transcriptome based protein sets of *M.* sp. CCMP2099 and *D. tenuilepis* contain 452 and 435 of the CGreenCut2, or 88 and 84 %, respectively. It is possible that GreenCut2 genes are more highly expressed than the average gene. This would lead to better transcript coverage, which then could result in an overrepresentation of GreenCut2 proteins in our transcriptome-based sets. Indeed, in our CCMP1545 and RCC299 short read samples, only 4 % of GreenCut2 genes were found in the 10 % of genes with the lowest expression. This indicates that the transcriptome based protein sets lack at least 12 and 16 % of the proteomes, respectively.

### Functional annotation and pathway finding

Interproscan v5 [21] was used with default settings and including the PANTHER protein set [99] to functionally annotate the predicted proteins of CCMP1545, RCC299, CCMP2099 and *D. tenuilepis*. TargetP was used to predict subcellular localization of peptidoglycan pathway and *A. thaliana* GreenCut2 proteins [100]. For CCMP1545 and RCC299 additional functional annotations were performed using the JGI Annotation Pipeline [101] in addition to manual annotations. For these two taxa the final genome builds, model sets and functional annotations are available on public genome browsers at portals at http://genome.jgi.doe.gov/Micromonas_pusilla/ and http://genome.jgi.doe.gov/Micromonas_commoda/.

### Phytozome gene families

*P. patens* PG pathway proteins [58, 60] were blasted (score cutoff 10^−15^) against the Viridiplantae ancestor node in Phytozome version 11 at https://phytozome.jgi.doe.gov/pz/portal.html. Phytozome gene families were found for all PG pathway proteins except PBP. One family (#63999465) contained the PBP transpeptidase domain (Pfam 00905) but not the transglycosylase domain (Pfam 00912) necessary for full PBP function. When more than five but less than all nine of the biosynthesis pathway proteins were found in an organism, tblastn (E-value ≤10^−15^) was attempted against the genome with the missing proteins as queries.

To find additional shared orthologs in the organisms that contained the full PG pathway, species information for all Phytozome v9.1 gene families were downloaded and filtered to remove families that contained taxa with only the four PG core genes and those containing fewer than eight species. This analysis was done with Phytozome v9.1 gene families instead of Phytozome v11 because v11 gene clusters are not available for download. The v9.1 gene clusters are available for download from Phytozome in the file: “global_analysis/families/cluster_members_Viridiplantae_3437.tsv”. Of the remaining 26 gene families, 19 were PG pathway proteins, one was a galactosyltransferase, one contained the NB-ARC domain and five contained a LysM protein.

The five LysM domain families were all subsets of family #38874692, comprising 14 proteins from 10 organisms. The proteins were scanned using TMHMM [102] and 13 were found to have N-terminal transmembrane domains. These 13 proteins were then used as input to the meme motif finder [96] with parameters -protein -mod zoops -nmotifs 2 -minsites 10 -maxsites 24 -minw 18 -maxw 25. Two motifs were found, one of which overlapped the LysM domain and the other matching the transmembrane regions. The meme suite program fimo [97] was used to match the two motifs against all Phytozome version 9.1 proteins using --output-pthresh 1e-10. Proteins were retained only when they contained both domains, resulting in a total of 14 proteins from 10 organisms, all of which contained the full PG pathway. This gene family is present as #63760547 in Phytozome 11, containing 18 genes in 13 organisms, all containing the full PG pathway. Fimo was run separately against databases of *Micromonas*, *D. tenuilepis,* and red algal proteins [39], but no matches were found.

### Phylogenetic reconstructions

Archaeplastida plastid sequences used in [103] were amended with additional prasinophyte and streptophyte homologs from GenBank, the Chloroplast Genome Database (CpBase, http://chloroplast.ocean.washington.edu/) and the Marine Microbial Eukaryote Transcriptome Sequencing Project [39]. Sixteen conserved plastid-encoded proteins were present in the partial chloroplast genome sequence from CCMP1545 (rpoC2, rpo1B, rpoC1, psaA, psbA, psaB, psbF, psbL, rpl14, rpl16, rps11, rps18, rps19, rps3, rps7, and rps8) and this subset was used to infer relationships across a total of 45 Archaeplastida taxa. First, protein sequences were aligned with MAFFT [104], the alignments were refined with the ED program implemented in MUST and regions of unambiguous alignment eliminated [105], as were positions having gaps. Absent proteins were treated as missing data. The individual protein alignments were then concatenated to a single alignment comprising 5,286 amino acid positions and analyzed using Maximum Likelihood (ML) methods (under the cpREV + G model and 5 rate categories, [106]) in PhyML [107]. Node support was computed with 1000 bootstrap replicates. The tree was rooted with the glaucophyte *Cyanophora paradoxa* as outgroup for display purposes.

For peptidoglycan protein reconstructions, amino acid sequences were retrieved from Phytozome 11 for streptophytes and MMETSP for prasinophytes and the glaucophyte. Besides the PPASP dataset, 2 examples of PG pathway proteins present throughout the Viridiplantae (MURE) or distributed in only some clades (MURA) were used to illustrate their evolutionary histories. Sequences were aligned with MAFFT and positions selected by Gblocks with default parameters [108]. The ML trees were built with FastTree [109] using the standard implementation GTR + CAT with 20-parameter gamma optimisation and a mix of nearest-neighbor interchanges and sub-tree-prune-regraft for the topology search. ML tree branch supports were analysed using Shimodaira–Hasegawa test values with 1000 replicates.

### Antibiotic experiments and coulter counter size measurements

RCC299 and CCMP1545 were grown under a 14 h/10 h light/dark cycle in L1 media in artificial seawater (as above) at 220 μE m^−2^ s^−1^ PAR. Both strains were maintained in light-acclimated, mid-exponential growth before experiment initiation. Two days before the experiment start cultures of each species were split into duplicates A and B. Ten mM Penicillin G (final concentration, i.e., 6000 Units ml^−1^) and 10 mM Fosfomycin (Sigma-Aldrich) were added 1 h after lights on (at T_0_). At each time point cells were fixed in 0.25 % glutaraldehyde (final concentration) for 30 min in the dark and frozen in liquid N_2_. Cells were measured using an Influx flow cytometer (BD Biosciences) and analyzed using Winlist (version 7.1, Verity Software House). Forward angle light scatter (FALS) and SSC were normalized using 0.75 μm diameter YG beads (Polysciences Inc.) and chlorophyll fluorescence (692 ± 40 nm band pass) was normalized to 2 μm diameter Polychromatic Red beads (Polysciences, Inc.).

To measure RCC299 for the morphological description, >10,000 cells from a mid-exponential phase, axenic culture were measured live on a Coulter Multisizer II approximately midway through the light period. Cells were grown on a 14 h/10 h light/dark cycle in K medium in artificial seawater maintained in mid-exponential growth for >10 generations after acclimatization to 21 °C and 90 μmol photons m^2^ sec^−1^ PAR.

## Additional files

Additional file 1: Table S1. Average GC%, size, protein-encoding gene count and density per *M. pusilla* CCMP1545 chromosome. Finished chromosomes are indicated. **Table S2.** Ortholog similarity in prasinophytes. The gene fraction is the ortholog count divided by the number of proteins in the smaller set. **Table S3.** OrthoGroups numbers across the Class II prasinophytes (see also Fig. 4) and number of OrthoGroups with >1 protein from the respective taxon in this analysis. Note that the higher redundancy of transcriptome-based proteomes of *D. tenuilepis* and CCMP2099 is likely due to artefacts of the method used. **Table S4.** Predicted proteins present in *Micromonas* and *Dolichomastix*, but not in *Bathycoccus* or *Ostreococcus*. **Table S5.** Genes encoding proteins in the peptidoglycan biosynthesis pathway identified here in prasinophytes, streptophytes and glaucophytes. Gene IDs from the new annotation generated here as shown in the JGI system (CCMP1545, RCC299, *Ostreococcus* RCC809), CAMPEP (CCMP2099, *D. tenuilepis*, *N. pyriformis*, *P. salinarum*, *G. wittrockiana,* see www.iplantcollaborative.org), Genbank (*O. tauri*, *O. lucimarinus*, and *B. prasinos*) and Phytozome version 11 (all others). tblastn: Gene found using tblastn against the genome (streptophytes) or transcriptome (CCMP2099, *D. tenuilepis*), no corresponding gene model exists. **Table S6.** EST support for Viridiplantae peptidoglycan genes (tblastn with predicted peptide, E-value cutoff 10^−15^). A maximum of five ESTs are listed for each. **Table S7.** Signal peptides on Viridiplantae peptidoglycan pathway enzymes found using TargetP. C: Chloroplast; M: Mitochondrion; S: secreted; −: no signal; n/a: protein was located by tblastn in the genome and no corresponding gene model exists. **Table S8.** Greencut2 family proteins in prasinophytes. (XLSX 167 kb) Additional file 2: Figure S1. Percentage of GC nucleotides along the LGC of CCMP1545 (left top) and RCC299 (left bottom) and a typical non-LGC chromosome of each (right). GC fraction was calculated using a 30 kb sliding scale. **Figure S2**. Size distribution of prasinophyte predicted proteins. Transcriptome-based proteins are overrepresented in the shorter bins indicating likely assembly issues, especially in *D. tenuilepis*. **Figure S3**. PPASP contains a transmembrane domain (yellow box) and a LysM domain (red box), predicted to be on the outside of the membrane. Sequence logo motif generated with 14 input sequences. **Figure S4**. Phylogenetic analysis of MURA **(A)**, PPASP **(B)** and MURE **(C)** protein sequences. **Figure S5**. Cellular localization of *Micromonas* RCC299 and CCMP1545 protein families in the Ancestral and Core (essential) sets based on TargetP analysis. Mixed: members of an ortholog group show different targeting signals. (PDF 746 kb)

## Abbreviations

Aa: amino acid
bp: base pairs
COP: convergent overlapping pair
IE: introner element
kb: kilobases
LC-MS: liquid chromatography tandem mass spectrometry
LGC: low-GC region
nt: nucleotides
ORF: open reading frame
PBP: penicillin binding protein
PG: peptidoglycan
PPASP: peptidoglycan pathway associated Streptophyte protein
RSI: regular spliceosomal intron
SLR: Sel-1 like repeat
