# Supplementary material for: Evidence-based green algal genomics reveals marine diversity and ancestral characteristics of land plants
Source: BMC Genomics. 2016 Mar 31;17:267. doi: 10.1186/s12864-016-2585-6 (PMC4815162; doi:10.1186/s12864-016-2585-6)
Supplement: Additional file 2: Figure S1. — Percentage of GC nucleotides along the LGC of CCMP1545 (left top) and RCC299 (left bottom) and a typical non-LGC chromosome of each (right). GC fraction was calculated using a 30 kb sliding scale. Figure S2. Size distribution of prasinophyte predicted proteins. Transcriptome-based proteins are overrepresented in the shorter bins indicating likely assembly issues, especially in D. tenuilepis. Figure S3. PPASP contains a transmembrane domain (yellow box) and a LysM domain (red box), predicted to be on the outside of the membrane. Sequence logo motif generated with 14 input sequences. Figure S4. Phylogenetic analysis of MURA (A), PPASP (B) and MURE (C) protein sequences. Figure S5. Cellular localization of Micromonas RCC299 and CCMP1545 protein families in the Ancestral and Core (essential) sets based on TargetP analysis. Mixed: members of an ortholog group show different targeting signals. (PDF 746 kb) [file 12864_2016_2585_MOESM2_ESM.pdf]

Supplemental Figures

Van Baren et al. Figure S1

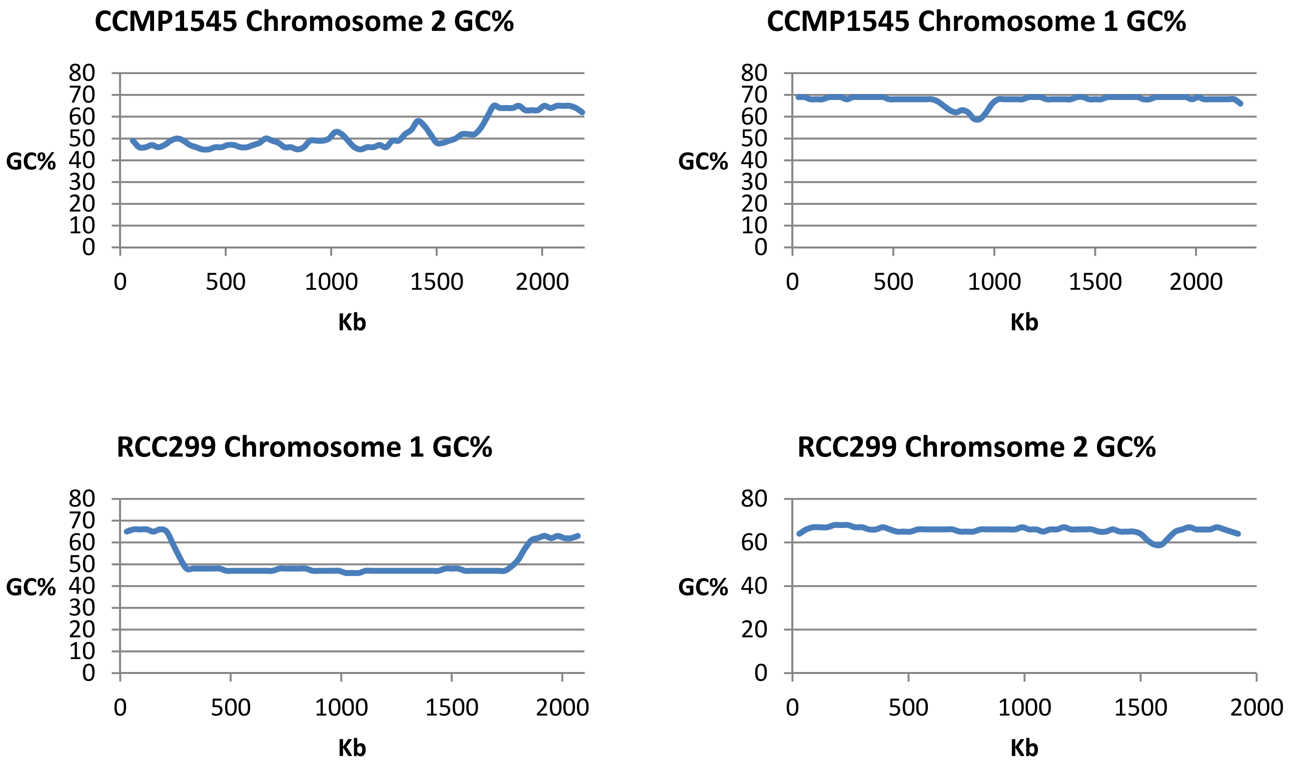

Van Baren et al. Figure S2

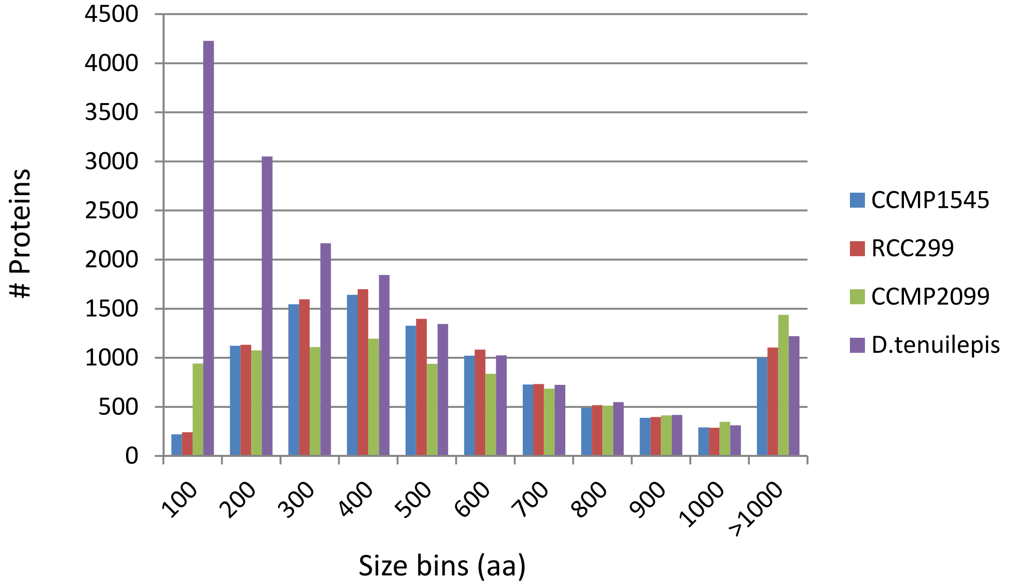

Van Baren et al. Figure S3

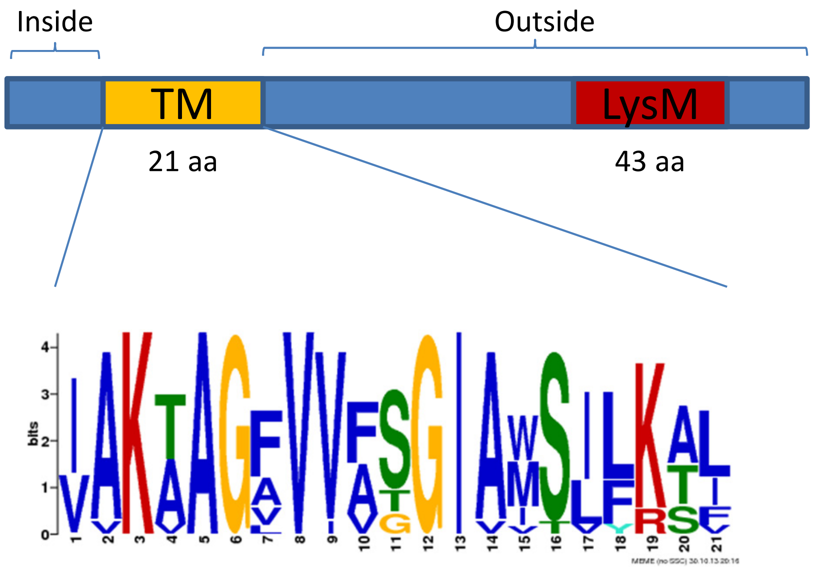

Van Baren et al. Figure S4

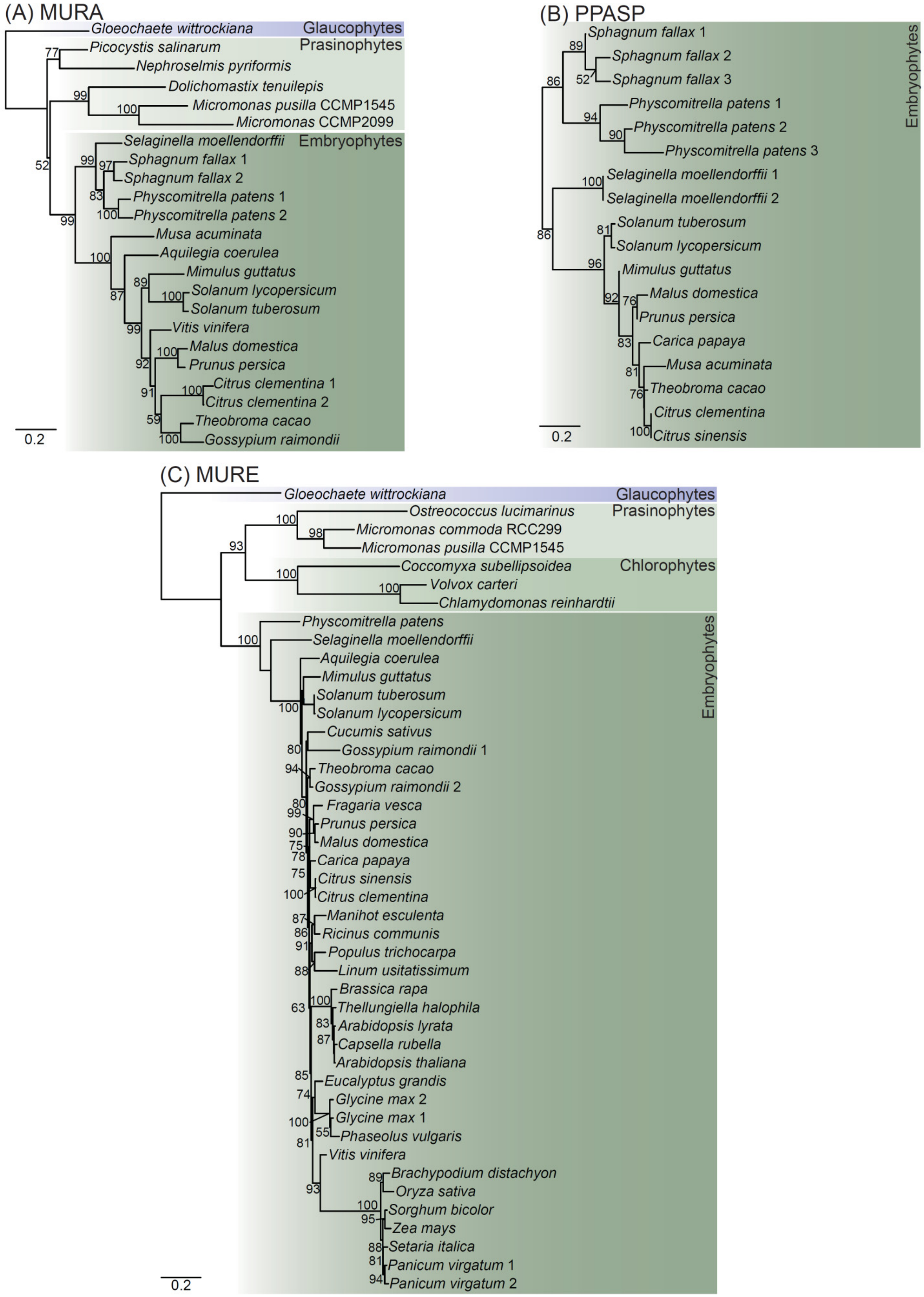

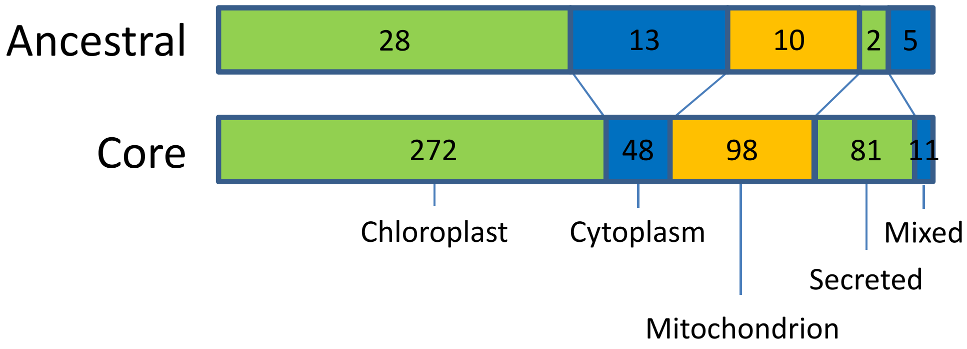
Ancestral
28
13
10
2
5
Core
272
48
98
81
11
Chloroplast
Cytoplasm
Mitochondrion
Secreted
Mixed
